# Synthesis of cyanooxovanadate and cyanosilylation of ketones†

**DOI:** 10.1039/d1ra05879g

**Published:** 2021-09-27

**Authors:** Yuji Kikukawa, Hiroko Kawabata, Yoshihito Hayashi

**Affiliations:** Department of Chemistry, Graduate School of Natural Science and Technology, Kanazawa University Kakuma Kanazawa 920-1192 Japan kikukawa@se.kanazawa-u.ac.jp

## Abstract

The cyanosilylation was performed by using metavanadate catalysts, and *in situ* measurements revealed the formation of [VO_2_(CN)_3_]^2−^ and [VO_4_TMS_2_]^−^ under reaction conditions. The reaction of [VO_2_(CN)_3_]^2−^, trimethylsilyl cyanide (TMSCN), and water afforded [VO_4_TMS_2_]^−^ and CN^−^, which reacted with ketones to yield the corresponding cyanohydrin trimethylsilyl ethers over [VO_2_(CN)_3_]^2−^. Compound [VO_2_(CN)_3_]^2−^ showed high catalytic performance for cyanosilylation of various carbonyl compounds. In the case of *n*-hexanal, turnover frequency reached up to 250 s^−1^.

## Abstract 2

Two key catalytic vanadium species involved in cyanosilylation of ketones were observed by *in situ* measurement.

## Graphical abstract

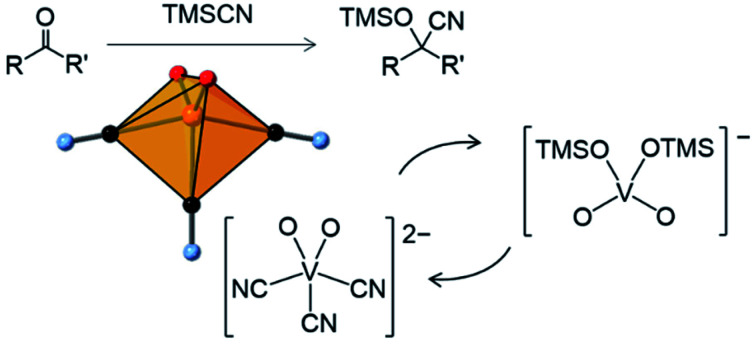

## 1. Introduction

Chemical reactions promoted by bases, such as isomerisation of alkenes and alkynes, formation of C–C, Si–C, and P–C bonds, nucleophilic ring opening of epoxides, and synthesis of heterocycles, are important for the production of several organic chemicals in both academic research and industrial processes.^1–3^ In some procedures, a stoichiometric amount (or more) of inorganic or organic bases is still used. Catalytic systems are favourable for environmental and energy sustainability. Inorganic solid base catalysts such as mixed oxides, zeolites, metal phosphates, and metal oxynitrides have been developed.^2^ However, it is difficult to construct uniform base sites in these catalysts. Recently, molecular metal oxide-based catalysts have received attention because they possess well-defined structures with tunable basicity, which enables to investigate the reaction mechanism with the elucidation of accurate active sites.^3^

Polyoxometalates are a large family of early transition metal oxide cluster anions. They have attracted research attention because of their applicability in different fields of science.^4^ Various polyoxometalates have been reported as oxidation and acid catalysts because their redox and acidic properties can be controlled by changing constituent elements and structures. The surfaces covered with oxygen atoms have a potential to act as Lewis base sites. In fact, several base catalysts of polyoxometalate have been reported over the last few decades and have been well summarised by K. Kamata.^3^ For the effective Lewis base reaction, the choice of a counter cation was important. Dilacunary polyoxometalates are inorganic multidentate ligands (Lewis bases) known to stabilise a variety of metal cores at its vacant sites.^5^ Non-protonated dilacunary silicotungstate [SiW_10_O_36_]^8−^ was isolated as a potassium salt at pH 9.1. However, the potassium cations strongly interact with its Lewis base sites.^6^ To improve the Lewis basic catalytic ability, the cation exchange with tetraalkylammonium, was reported to be effective.^7^ In the case of vanadium-based polyoxometalates (POVs), Lewis basicity to stabilise several metal cores is also observed with POVs composed of tetrahedrally coordinated VO_4_ units. The precursor [V_4_O_12_]^4−^ was synthesised under more basic conditions than [SiW_10_O_36_]^8−^.^8^ [VO_4_]^3−^ has a larger p*K*_a_ value than [WO_4_]^2−^ and is expected to exhibit higher base catalytic performance than tetrahedral monometalates.^9^ Unlike tungsten-based polyoxometalates, POV structures are easily transformed upon chemical stimuli. Therefore, when the labile POVs are employed as a catalyst, *in situ* analysis of vanadium species under catalytic conditions is indispensable to understand a true mechanism of catalytic reactions.

Herein, cyanosilylation reaction of ketones by a POV catalyst was investigated in this study. As cyanohydrins are utilised as important intermediates, the development and understanding for the cyanosilylation of carbonyl compounds is important.^10^

## 2. Results and discussion

**Scheme 1:**
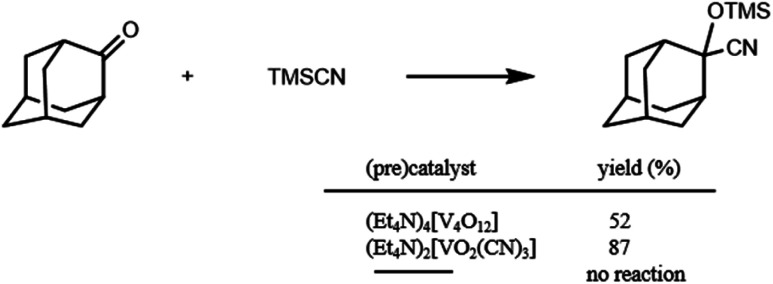
Cyanosilylation of 2-adamantanone with TMSCN. Reaction conditions: catalyst (0.2 mol% based on vanadium), 2-adamantanone (1 mmol), TMSCN (1.2 mmol), acetonitrile 1 mL, 32 °C, 15 min.

The cyanosilylation of 2-adamantanone with trimethylsilyl cyanide (TMSCN) was carried out with {Et_4_N}_4_[V_4_O_12_] in acetonitrile at 32 °C for 15 min. As shown in Scheme 1, cyanosilylation affords the corresponding cyanohydrin trimethylsilyl ether in 52% yield. In the absence of catalysts, cyanosilylation did not proceed under the present conditions. *In situ* spectroscopic measurements were performed to investigate a true reactive species. The positive-ion cold-spray ionisation mass (CSI-MS) spectrum of an {Et_4_N}_4_[V_4_O_12_] acetonitrile solution showed two sets of signals at *m*/*z* 817 and 1047, which were assigned to {(Et_4_N)_4_[V_3_O_9_]}^+^ and {(Et_4_N)_5_[V_4_O_12_]}^+^, respectively (Fig. S1†). These species gave ^51^V NMR resonances at −574 and −568 ppm with similar intensity ratios to that of the CSI-MS spectrum (Fig. S2†). These results show that metavanadate species, V_*x*_O_3*x*_*^x^*^−^, are under a trimer–tetramer equilibrium in acetonitrile.^8^ Upon the addition of TMSCN, the CSI-MS spectrum showed a set of signals at *m*/*z* 521 and 551, which were assigned to {(Et_4_N)_2_VO_4_TMS_2_}^+^ and {(Et_4_N)_3_VO_2_(CN)_3_}^+^; these species are attributed to the structural transformation that formed monomeric vanadium species (Fig. S1†). These species gave ^51^V NMR resonances at −706 and −672 ppm (Fig. S2†). These results indicated structural transformation to monomeric vanadium species and stimulated the synthesis of the reactive vanadium catalyst. We successfully obtained crystals of (Et_4_N)_2_[VO_2_(CN)_3_], which showed a resonance only at −672 ppm in the ^51^V NMR spectrum, from the solution under a higher vanadium concentration condition, whereas we did not succeeded in the isolation of (Et_4_N)_2_[VO_4_TMS_2_].

**Fig. 1:**
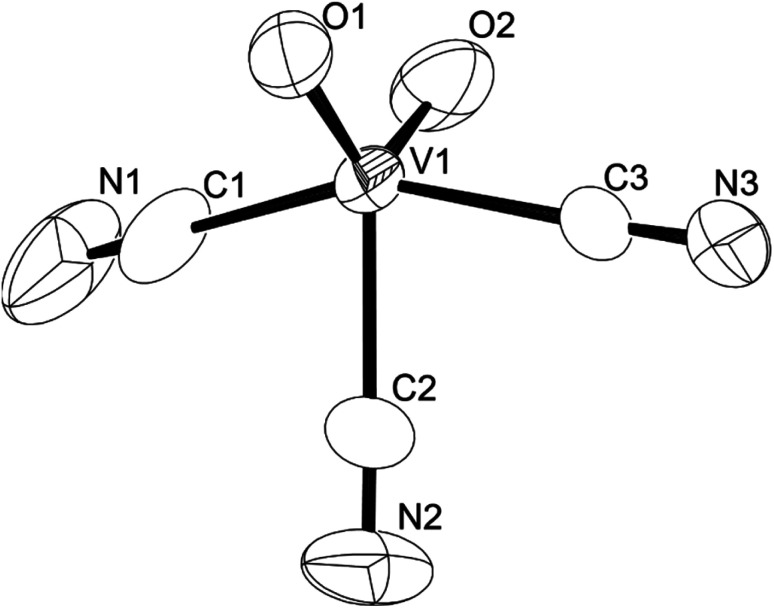
ORTEP representation of cyanooxovanadate, [VO_2_(CN)_3_]^2−^.

We established a reliable synthesis of cyanooxovanadate, [VO_2_(CN)_3_]^2−^, from a solution of (Et_4_N)_4_[V_4_O_12_] with 12 equiv. of {Et_4_N}CN and 8 equiv. of *p*-toluenesulfonic acid. The addition of an excess amount of ethyl acetate yielded a powder. Single crystals suitable for X-ray analysis were obtained by recrystallisation in a mixed solvent of acetonitrile and ethyl acetate (Table S1†).‡‡The UV spectrum of (Et_4_N)_2_[VO_2_(CN)_3_] in acetonitrile showed a band at 283 nm with *ε* = 2.6 × 10^3^ M^−1^ cm^−1^ (Fig. S5). The ORTEP representation of the anion is shown in Fig. 1. Cyanooxovanadate possesses a distorted trigonal bipyramidal coordination geometry. A similar vanadium coordination geometry was observed in some dioxovanadium complexes.^11^ The V–O distances were in the range of 1.605–1.628 Å, indicating the presence of non-protonated oxo ligands. The three cyano ligands were in the same plane. In addition, the middle cyano ligand and two oxo ligands were in the vertical plane. Thus, the anion possesses *C*_2v_ symmetry. Two tetraethylammonium counter cations per vanadium species were determined, suggesting that the valency of vanadium atoms is five. The positive-ion CSI-MS spectrum of this compound showed a signal only at *m*/*z* 551 (Fig. S3†). The IR spectrum showed absorbances at 2134 and 2144 cm^−1^ and 949 cm^−1^, corresponding to *ν*(C–N) and *ν*(V–O), respectively (Fig. S4†). Until now, cyanovanadates such as [V(CN)_6_]^4−^, [V(CN)_7_]^4−^, [V(CN)_6_NO]^4−^, and [VO(CN)_5_]^3−^ have been reported.^12^ To the best of our knowledge, cyanooxovanadate [VO_2_(CN)_3_]^2−^ has not yet been reported.

**Fig. 2:**
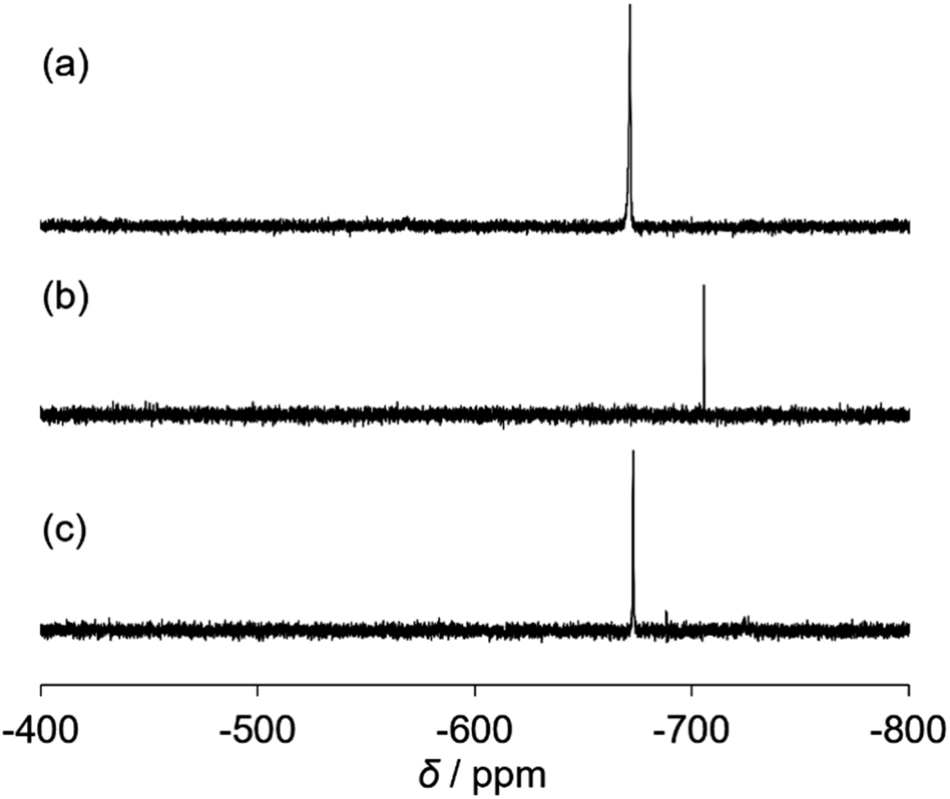
^51^V NMR spectra of the catalytic reaction solution (a) before, (b) during, and (c) after the reaction. Reaction conditions: (Et_4_N)_2_[VO_2_(CN)_3_] (1 μmol), 2-adamantanone (0.5 mmol), TMSCN (0.5 mmol), and acetonitrile (0.5 mL) in an NMR tube.

The catalytic properties of (Et_4_N)_2_[VO_2_(CN)_3_] were investigated. For the cyanosilylation of 2-adamantanone with TMSCN, the catalytic performance of (Et_4_N)_2_[VO_2_(CN)_3_] was higher than that of (Et_4_N)_4_[V_4_O_12_] (Scheme 1). The CSI-MS spectrum of the catalytic reaction solution showed a set of signals at *m*/*z* 521, owing to the formation of {(Et_4_N)_2_VO_4_TMS_2_}^+^ during the reaction (Fig. S3†). After the reaction, a set of signals at *m*/*z* 551 corresponding to {(Et_4_N)_3_VO_2_(CN)_3_}^+^ retrieved. The analysis of the ^51^V NMR spectra also indicated a change in the vanadium species. During the reaction, a resonance at −706 ppm was observed, and at the end of the reaction, this resonance disappeared, and a resonance peak at −673 ppm was retrieved, which corresponded to (Et_4_N)_2_[VO_2_(CN)_3_] (Fig. 2).

**Fig. 3:**
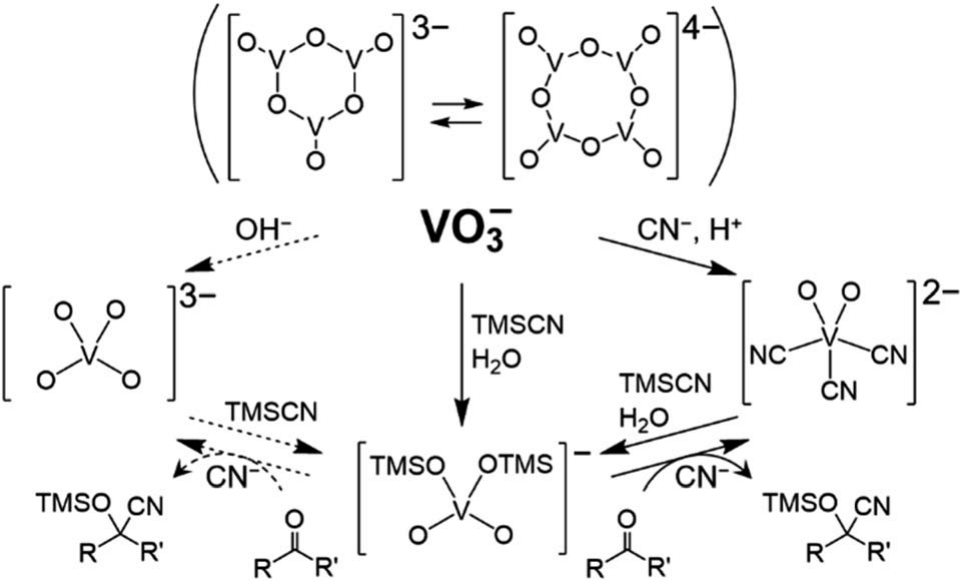
Relationship among vanadium–oxo complexes. Dotted lines are expected reactions involving [VO_4_]^3−^.

The intriguing fact is the formation of [VO_4_TMS_2_]^−^ (Fig. 3). The nucleophilic activation of TMSCN by [VO_2_(CN)_3_]^2−^, followed by the reaction with water (over the catalyst and/or in the presence of the solvent), led to the formation of (Et_4_N)[VO_4_TMS_2_]. Compound [VO_4_TMS_2_]^−^ was also expected to be formed in the reaction of [VO_4_]^3−^, which can be formed by the addition of a Brønsted base to [V_4_O_12_]^4−^. Compound [VO_4_]^3−^ is expected to act as a strong base catalyst. Interestingly, [VO_4_TMS_2_]^−^ was formed from (Et_4_N)_2_[VO_2_(CN)_3_], which was synthesised by the addition of acid to [V_4_O_12_]^4−^ with Et_4_NCN. In addition, despite the identical formation of (Et_4_N)[VO_4_TMS_2_] during the reaction, (Et_4_N)_4_[V_4_O_12_], the precatalyst, exhibited a lower performance than (Et_4_N)_2_[VO_2_(CN)_3_]. From a stoichiometric viewpoint, a 2 cyanide ions with respective to  a vanadium atom are formed from (Et_4_N)_4_[V_4_O_12_] and 5 cyanide ions are formed from (Et_4_N)_2_[VO_2_(CN)_3_], suggesting that the amount of cyanide ions is essential for the reaction (see description below).

The following mechanism of ketone cyanosilylation was proposed: first, the cleavage of TMSCN by [VO_2_(CN)_3_]^2−^ afforded [VO_4_TMS_2_]^−^. Next, the nucleophilic attack of CN^−^ on the carbonyl group and transfer of the trimethylsilyl group produced cyano silyl ether.

In the presence of 0.2 mol% of (Et_4_N)_2_VO_2_(CN)_3_, various types of structurally diverse ketones can be converted to the corresponding cyanohydrin trimethylsilyl ethers without the formation of desilylated products (cyanohydrins) (Table 1). The cyanosilylation of cyclic and open chain ketones proceeded with high efficiency. Cyclohexanone was more reactive than cyclopentanone. Sterically demanding ketones required longer reaction time. Although the α,β-unsaturated ketone of 2-cyclohexen-1-one was less reactive, the 1,2-addition of TMSCN took place to afford 1-cyano-1-trimethylsilyl-2-cyclohexene without a 1,4-addition (Michael addition) product. In addition, the less-reactive acetophenone derivatives were converted to the corresponding cyanohydrin trimethylsilyl ethers. The cyanosilylation of acetophenone derivatives with electron-withdrawing substituents resulted in higher reaction rates. In contrast, acetophenone derivatives with electron-donating substituents are less reactive. This indicated that the rate-determining step was the cyanide ion nucleophilic attack on the ketone carbon atom. The conversion of [VO_2_(CN)_3_]^2−^ to [VO_4_TMS_2_]^−^ produces protons *via* hydrolysis, which hinders the nucleophilic attack of cyanide ions. Because the possible reaction mechanism *via* [VO_4_]^3−^ does not include proton dissociation, a higher catalytic performance due to the presence of [VO_4_]^3−^ is expected.^13^ Cyanosilylation of propiophenone proceeded efficiently. Benzophenone and cyclopropyl phenyl ketone were less reactive than acetophenone.

**Table 1:** Cyanosilylation of various ketones^a^

Entry	Substrate	Time/min	Product	Yield/%
1	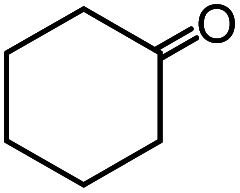	5	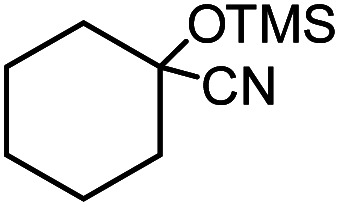	97
2	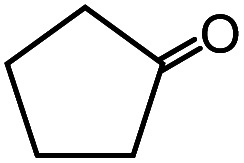	30	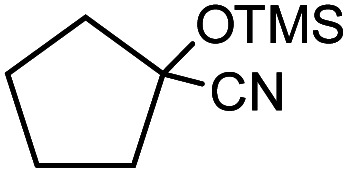	82
3	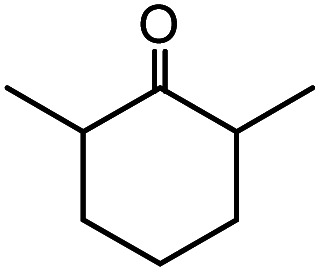	15	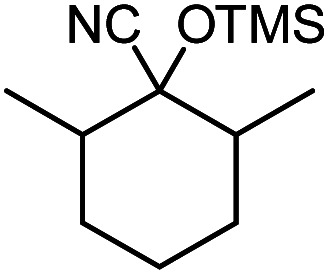	87
4	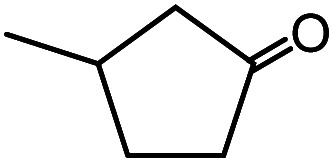	30	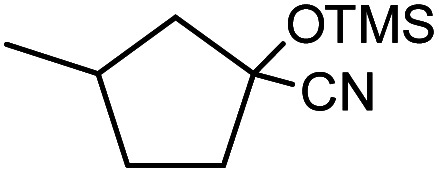	86
5	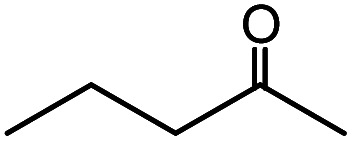	10	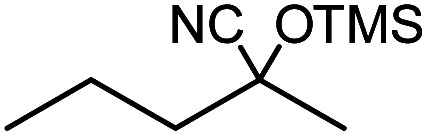	91
6	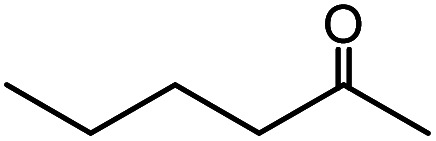	30	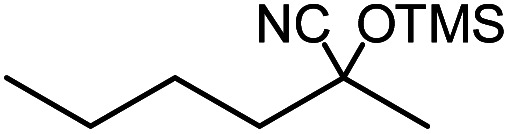	91
7	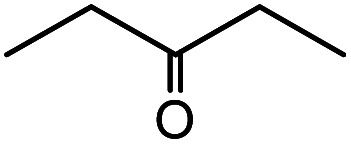	10	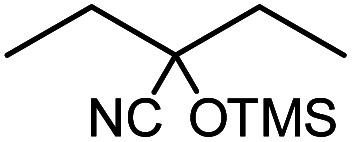	97
8	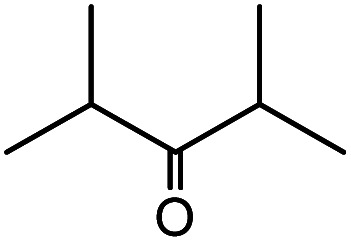	30	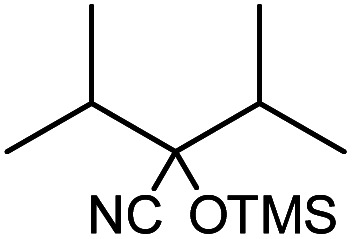	96
9	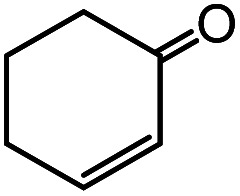	45	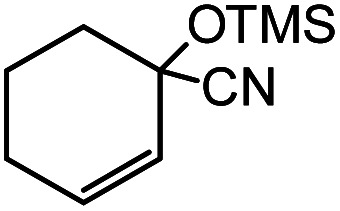	26
10	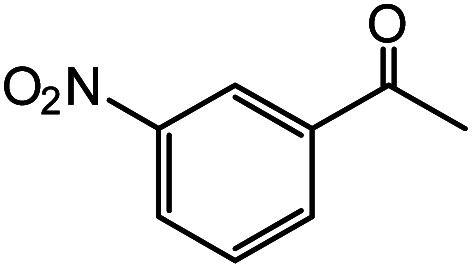	1	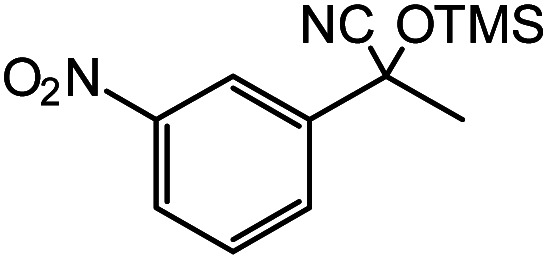	90^b^
11	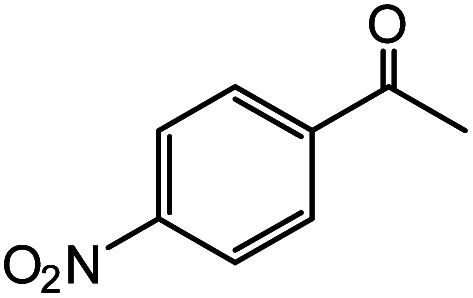	1	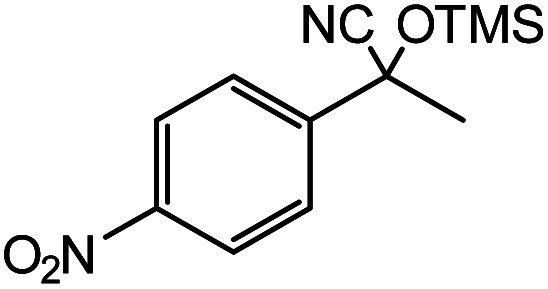	82^c^
12	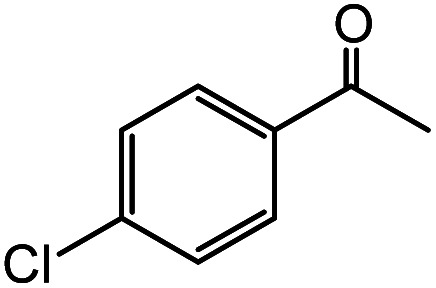	5	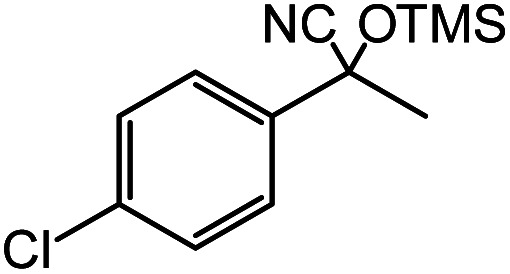	69
13	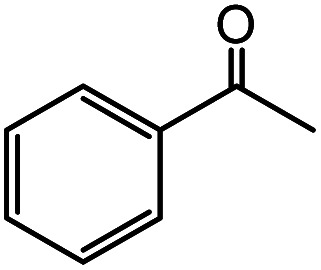	45	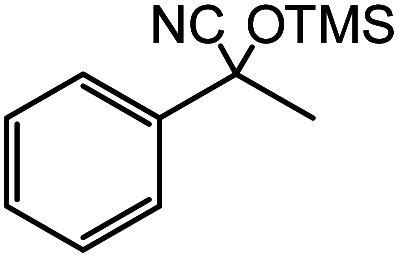	79
14	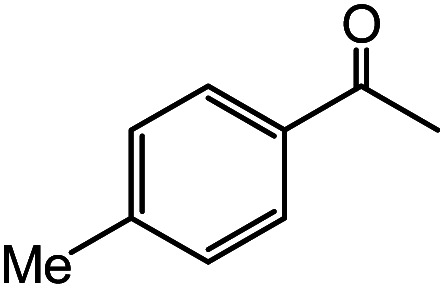	60	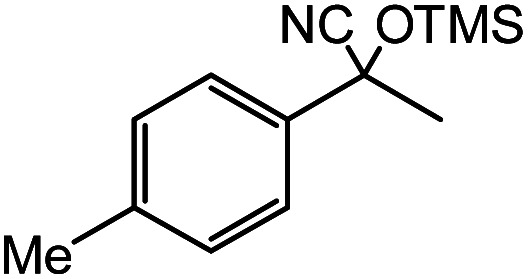	40
15	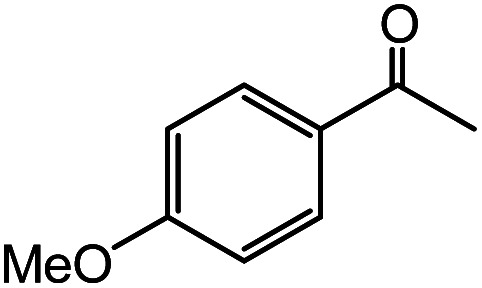	60	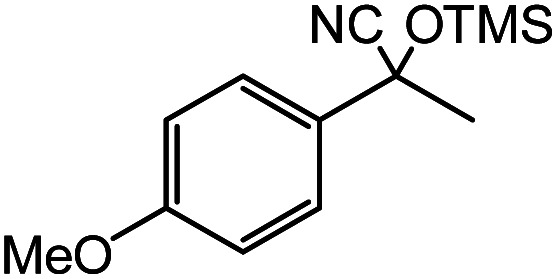	21
16	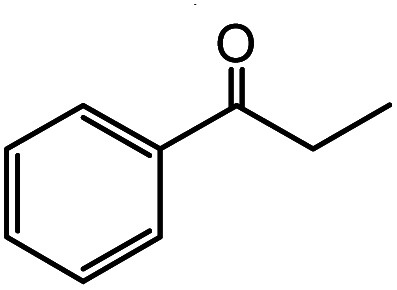	45	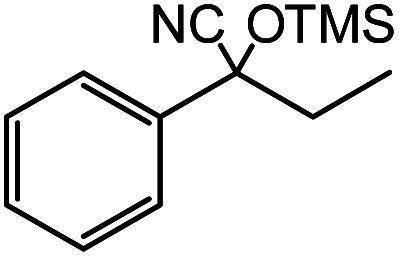	84
17	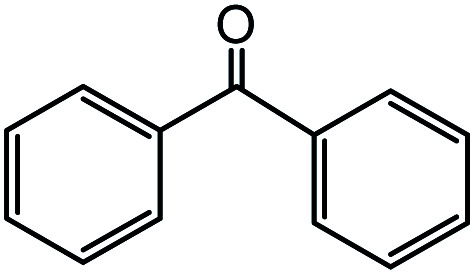	45	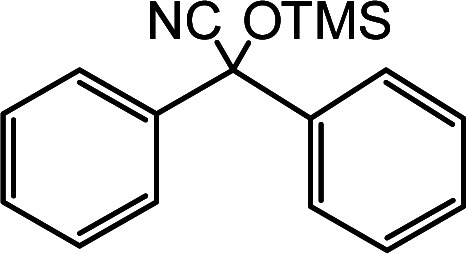	21
18	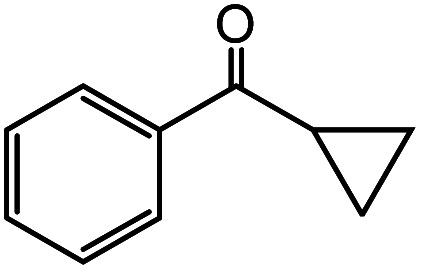	45	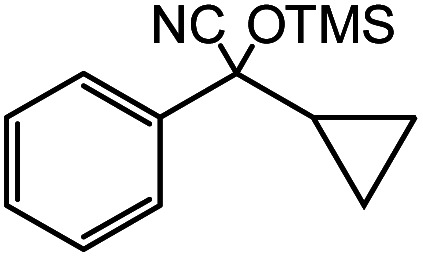	16

a Reaction conditions: {Et_4_N}_2_[VO_2_(CN)_3_] (2 μmol), substrate (1 mmol), TMSCN (1.2 mmol), acetonitrile (1 mL) at 32 °C. The yields were determined by GC using naphthalene as an internal standard.

b *ca.* 2% of unidentified product was detected.

c *ca.* 6% of unidentified product was detected.

**Scheme 2:**
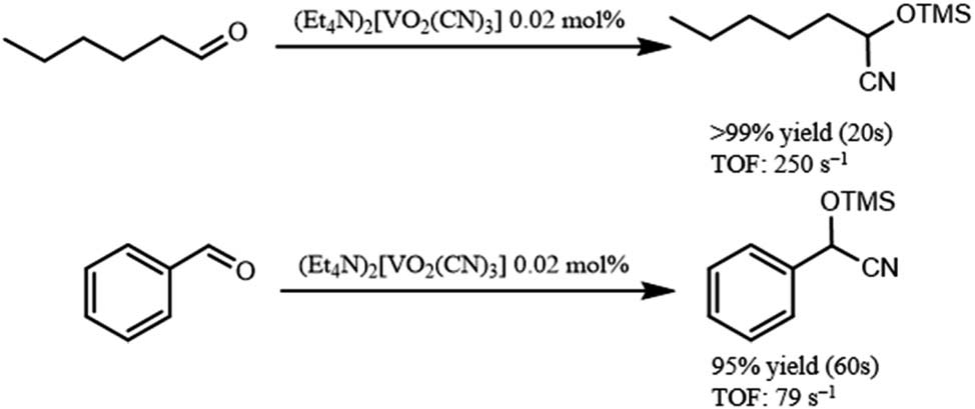
Cyanosilylation of aldehydes. Reaction conditions: {Et_4_N}_2_[VO_2_(CN)_3_] (0.2 μmol), aldehyde (1 mmol), TMSCN (1.2 mmol), acetonitrile (1 mL) at 32 °C. The yields were determined by GC using naphthalene as an internal standard.

In addition, (Et_4_N)_2_[VO_2_(CN)_3_] showed high catalytic performance for cyanosilylation of sterically less hindered aldehydes in comparison with ketones (Scheme 2). The cyanosilylation of *n*-hexanal with 0.02 mol% of (Et_4_N)_2_[VO_2_(CN)_3_] gave 2-trimethylsilanyloxy-heptanenitrile in >99% yield for 20 s. The turnover frequency (TOF) reached up to 250 s^−1^. In the case of benzaldehyde, cyanosilylation also proceeded with high TOF (79 s^−1^). These values are the highest level among the previously reported systems.^7^

## 3. Experimental

### Instruments

The cold-spray ionization mass spectra were recorded on a JEOL JMS-T100CS. NMR spectra were recorded with a JEOL JNM-LA400. ^51^V NMR spectra were measured at 105.15 MHz. Chemical shifts were referenced to VOCl_3_ (*δ* = 0 ppm) for ^51^V NMR. IR spectra were measured on a Jasco FT/IR-4100 using a nujol method. Elemental analyses of C, H, and N were performed by the Research Institute for Instrumental Analysis at Kanazawa University. GC analysis was performed on a Shimadzu GC-2014 with a flame ionization detector (FID) equipped with a ZB-WAXplus capillary column (phenomenex, internal diameter = 0.25 mm, length = 30 m), NEUTRABOND capillary column (GL science, internal diameter = 0.25 mm, length = 30 m), and ZB-5 capillary column (phenomenex, internal diameter = 0.25 mm, length = 30 m). Naphthalene was used as the internal standard. GC-MS spectra were measured on a Shimadzu GCMS-QP2010. UV/vis spectra were measured on a Jasco V-770.

### Reagents

Solvents and reagents were obtained from Wako and TCI. All chemicals were used as received. (Et_4_N)_4_[V_4_O_12_] was synthesized by the reported procedures.^8^ The authentic catalytic reaction products (cyanohydrin trimethylsilyl ethers) were prepared by the previously reported systems.^7,13^

### X-ray crystallographic analysis

Single crystal structure analysis was performed at 90 K with a Bruker D8 VENTURE diffractometer with Cu-Kα radiation (*λ* = 1.54178 Å). The data reduction and absorption correction were completed using the APEX3 program.^14^ The structural analyses were performed using APEX3 and winGX^15^ for Windows software. The structures were solved by SHELXT (direct methods) and were refined by using SHELXL-2014.^16^ Non-hydrogen atoms were refined anisotropically. Hydrogen atoms were positioned geometrically and were refined using a riding model. Crystallographic data are summarized in Table S1.† CCDC 2094039† contains the supplementary crystallographic data for this paper. These data can be obtained free of charge from The Cambridge Crystallographic Data Centre.

### Synthesis of (Et_4_N)_2_[VO_2_(CN)_3_]

All reactions and manipulations were conducted under a nitrogen atmosphere. (Et_4_N)_4_[V_4_O_12_]·H_2_O 100 mg (0.107 mmol) was dissolved in 2 mL of acetonitrile. (Et_4_N)CN 200 mg (1.28 mmol) and *p*-toluenesulfonic acid monohydrate 163 mg (0.856 mmol) were added to the solution with stirring. Addition of 10 mL of ethyl acetate to the solution yielded precipitates. The precipitates were collected by filtration and dried (67 mg, 37% yield based on vanadium). 100 mg of the crude powder was dissolved in 2 mL of acetonitrile, and addition of ethyl acetate gave 32 mg of colorless crystals. Anal. Calcd for (Et_4_N)_2_[VO_2_(CN)_3_]: C, 54.14; H, 9.57; N, 16.62; found: C, 53.91; H, 9.39; N, 16.41. IR (nujor; 2500–400 cm^−1^): 2143, 2134, 1482, 1458, 1395, 1377, 1362, 1305, 1186, 1174, 1119, 1081, 1071. 1053, 1–34, 1000, 949, 923, 898, 841, 789, 722, 661, 596, 457, 416 cm^−1^. ^51^V NMR (acetonitrile): *δ* = −672 ppm. UV (acetonitrile): 283 nm, *ε* = 2.6 × 10^3^.

### Cyanosilylation

The detailed reaction conditions are shown in the captions of Table and Schemes. A typical procedure for cyanosilylation of carbonyl compounds is as follows: into a pyrex-glass screw cap vial (volume: *ca.* 20 mL) were successively placed (Et_4_N)_2_[VO_2_(CN)_3_] (0.2 mol%), substrate (1 mmol), naphthalene (0.2 mmol) as internal standard and acetonitrile (1 mL). A Teflon-coated magnetic stir bar was added, and the reaction was initiated by addition of TMSCN (1.2 mmol). The reaction mixture was vigorously stirred (800 rpm) at 32 °C in 1 atm of air. The conversion and the product yield were periodically determined by GC analysis. All products were confirmed by comparison of their GC retention times and GC-MS with those of authentic data.

## 4. Conclusions

We monitored the cyanosilylation with a metavanadate catalyst through ^51^V NMR studies as well as CSI-MS spectroscopy, and the formation of a catalytic species, [VO_2_(CN)_3_]^2−^, was detected *in situ*. To confirm the catalytic ability of a tricyanodioxovanadate, it was isolated, characterized and proved to show a better performance in the catalytic reaction. In the catalytic cycle, ditrimethylsiloxydioxovandate was also produced and we have elucidated the two key catalytic species involved in each step of the cyanosilylation reaction.

## Conflicts of interest

There are no conflicts to declare.

## Supplementary Material

RA-011-D1RA05879G-s001

RA-011-D1RA05879G-s002

RA-011-D1RA05879G-s003
