# Supplementary material for: Synthesis of cyanooxovanadate and cyanosilylation of ketones
Source: RSC Adv. 2021 Sep 27;11(50):31688–92. doi: 10.1039/d1ra05879g (PMC9041443; doi:10.1039/d1ra05879g)
Supplement: RA-011-D1RA05879G-s001 [file RA-011-D1RA05879G-s001.pdf]

Electronic Supplementary Material (ESI) for RSC Advances.
This journal is © The Royal Society of Chemistry 2021

Supplementary information for

Synthesis of Cyanooxovanadate and Cyanosilylation of Ketones

Yuji Kikukawa,* Hiroko Kawabata and Yoshihito Hayashi

Department of Chemistry, Graduate School of Natural Science and Technology, Kanazawa University, Kakuma, Kanazawa 920-1192 (Japan)

Table S1. Crystallographic data for $(Et_4N)_2[VO_2(CN)_3]$

formula	$C_{57}H_{120}N_{15}O_6V_3$
fw	1264.49
crystal system	monoclinic
space group	$P2_1/c$ (#14)
a (Å)	8.2400(2)
b (Å)	34.3199(8)
c (Å)	12.7556(3)
β (deg)	93.1970(10)
V (Å^3)	3601.62(15)
Z	2
μ (mm^{-1})	3.616
R_1 ($I > 2\sigma(I)$)	0.0711
wR_2	0.1940

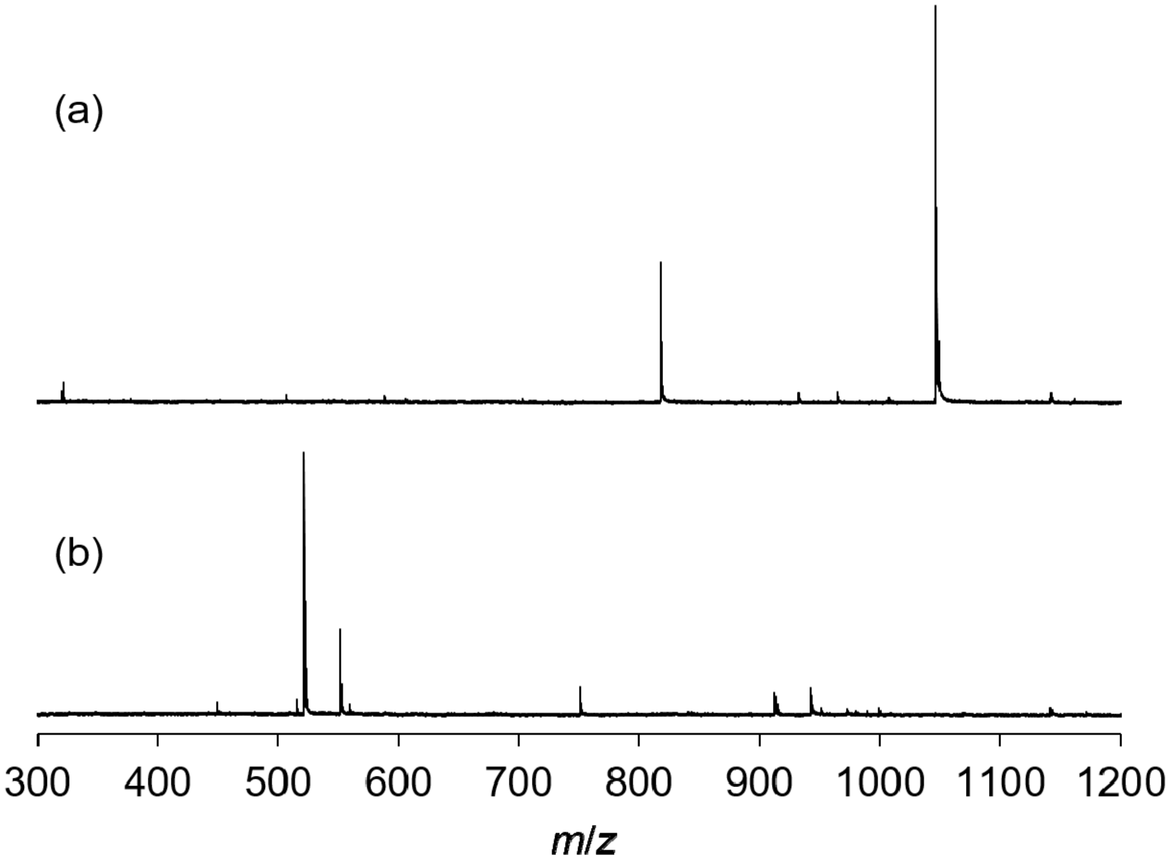

Figure S1. Positive mode of CSI-MS spectra of acetonitrile solution of tetraethylammonium salt of methavanadate (a) without addition of TMSCN and (b) in the presence of TMSCN. The main signal sets in (a) at m/z 817 and 1047 are assignable to $\{(Et_4N)_4[V_3O_9]\}^+$ and $\{(Et_4N)_5[V_4O_{12}]\}^+$, respectively. The main signal sets in (b) at m/z 521 and 551 are assignable to $\{(Et_4N)_2TMS_2VO_4\}^+$and $\{(Et_4N)_3VO_2(CN)_3\}^+$, respectively.

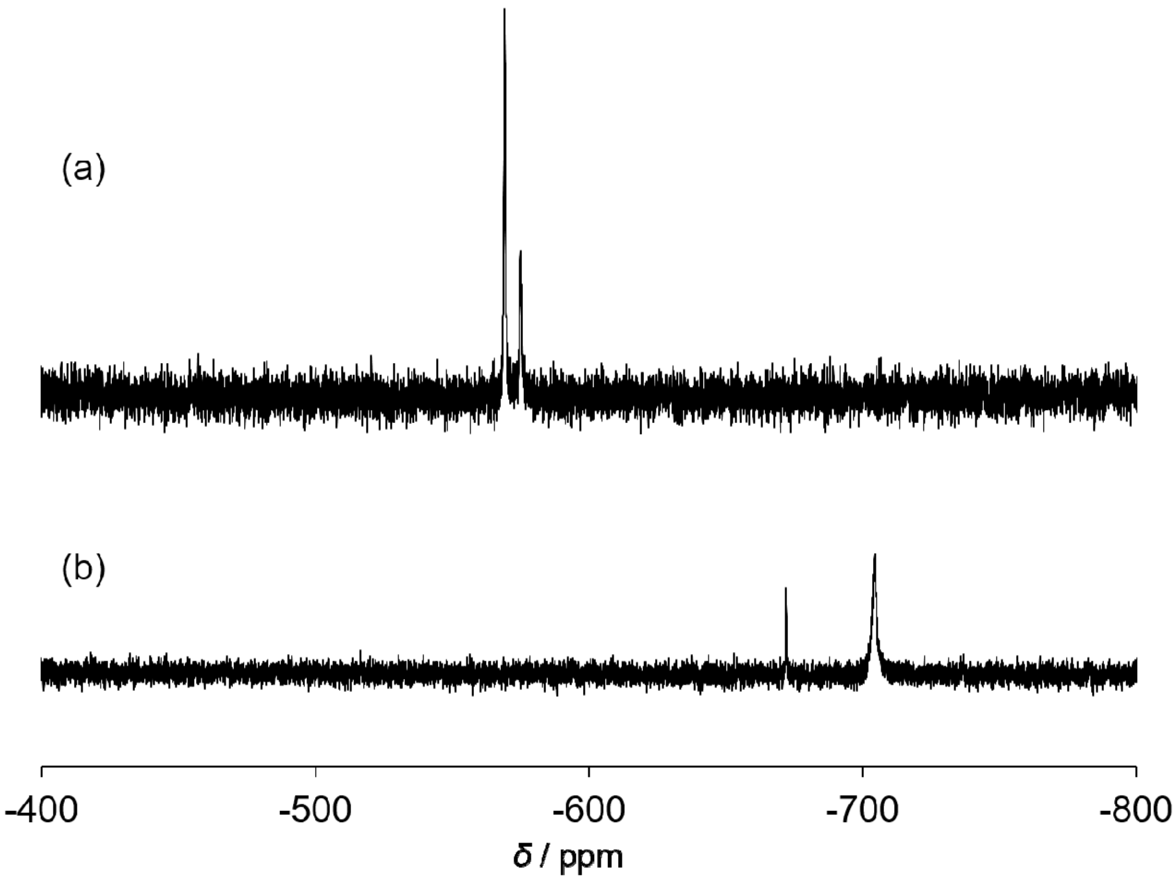

Figure S2. ^{51}V NMR spectrum of of tetraethylammonium salt of methavanadate (a) without addition of TMSCN and (b) in the presence of TMSCN. Vanadium concentration is 2 mM.

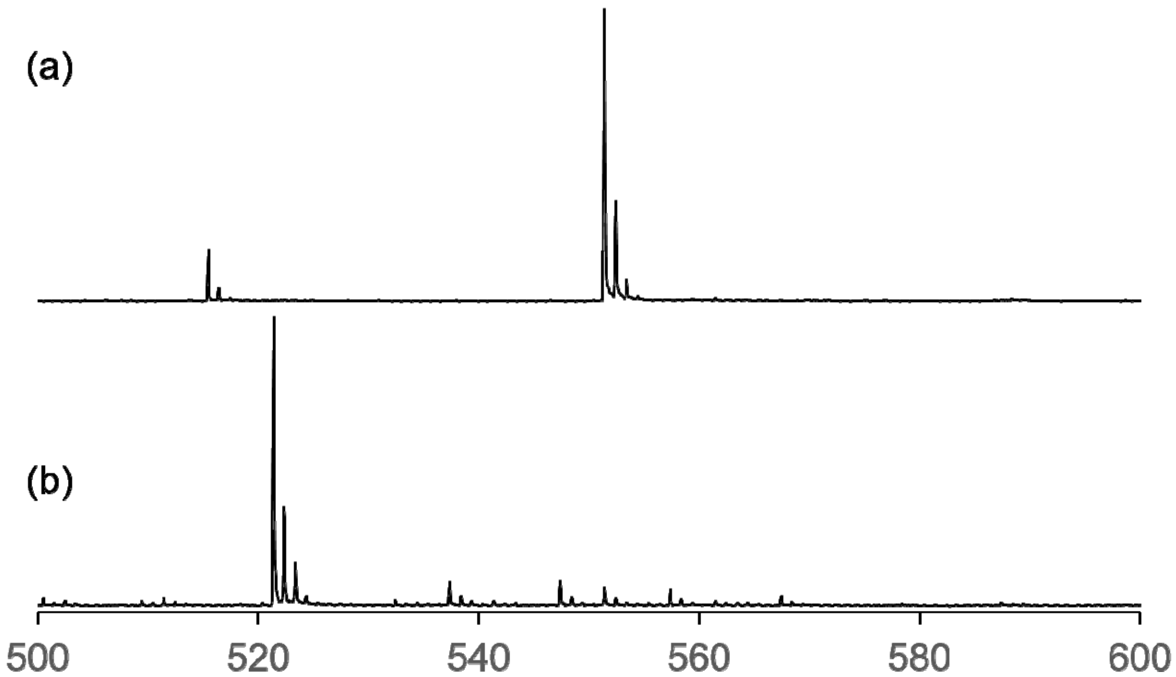

Figure S3. Positive mode of CSI-MS spectra of the reaction solution of cyanosilylation of 2-adamantanone with $(Et_4N)_2[VO_2(CN)_3]$ (a) before and (b) during the reaction. The main signal sets at m/z 551 in (a) and m/z 521 in (b) are assignable to $\{(Et_4N)_3VO_2(CN)_3\}^+$and $\{(Et_4N)_2TMS_2VO_4\}^+$, respectively.

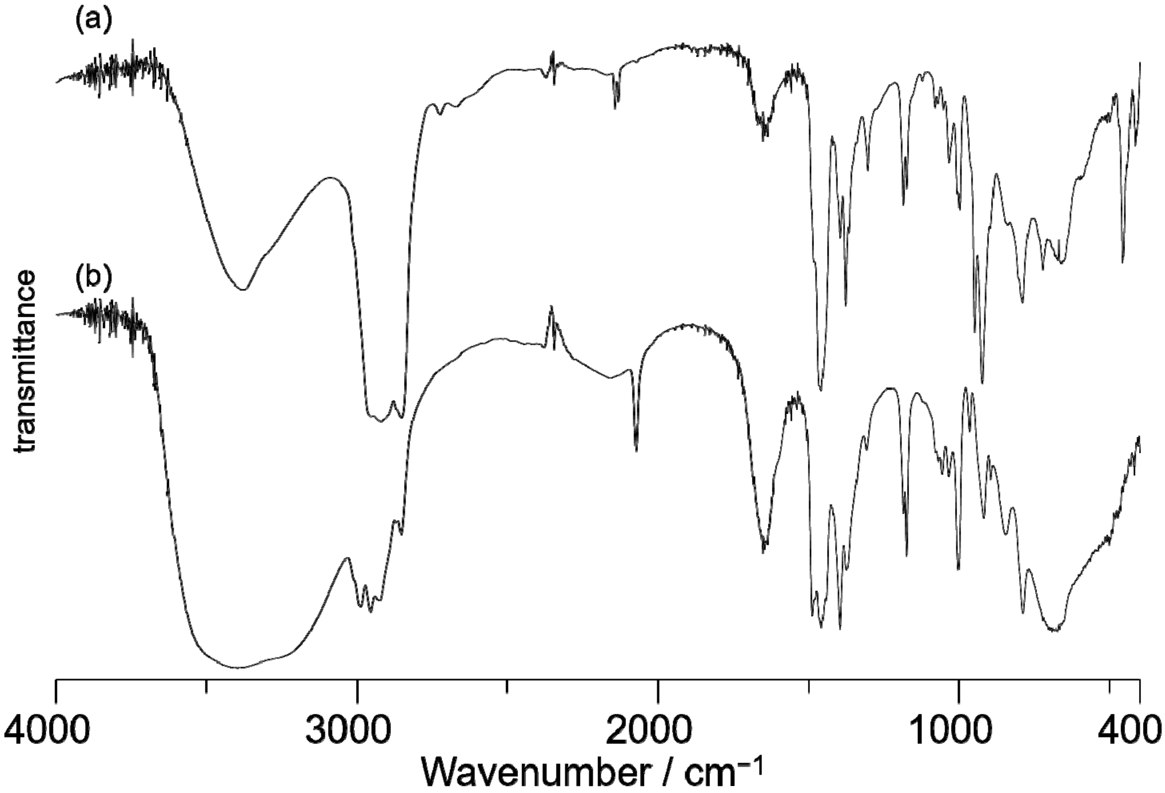

Figure S4. IR spectra of (a) $(Et_4N)_2[VO_2(CN)_3]$ and (b) Et_4NCN. Data are recorded with a nujor method.

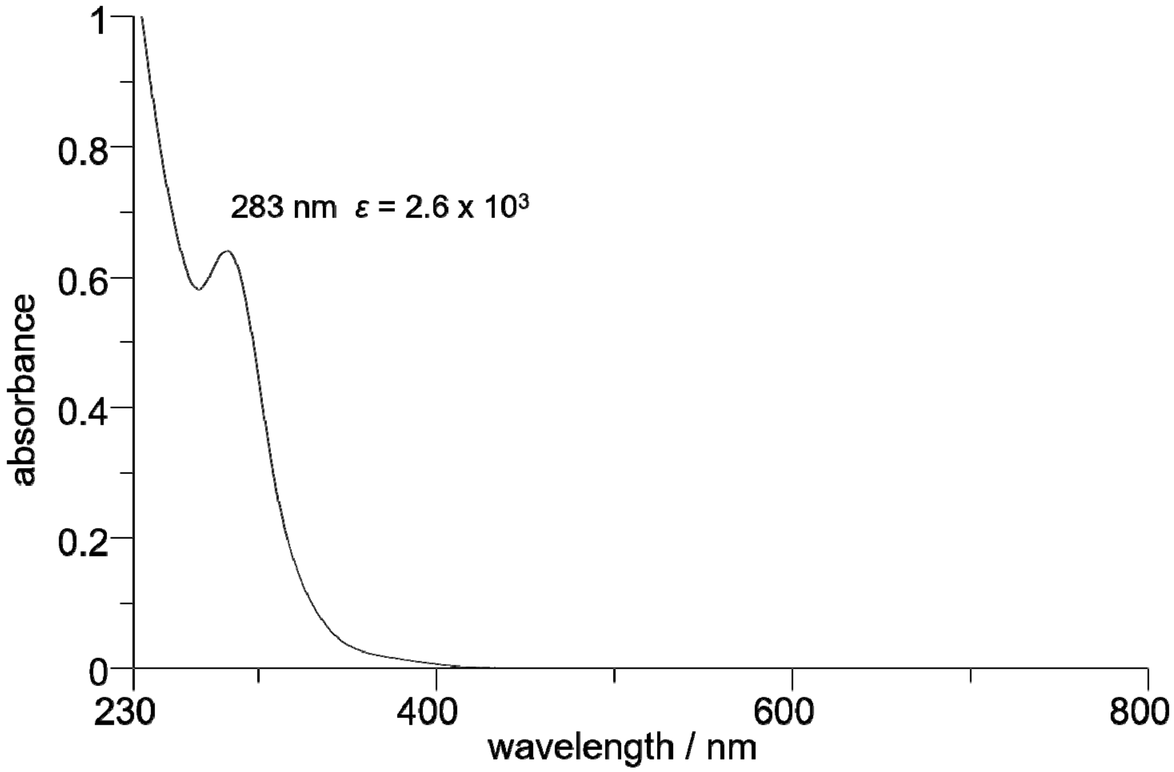

Figure S5. UV spectrum of $(Et_4N)_2[VO_2(CN)_3]$ in acetonitrile. The concentration of $(Et_4N)_2[VO_2(CN)_3]$ was 0.25 M.
