# Supplementary material for: Synthesis of cyanooxovanadate and cyanosilylation of ketones
Source: RSC Adv. 2021 Sep 27;11(50):31688–92. doi: 10.1039/d1ra05879g (PMC9041443; doi:10.1039/d1ra05879g)

Electronic Supplementary Material (ESI) for RSC Advances.
This journal is © The Royal Society of Chemistry 2021

GC chart

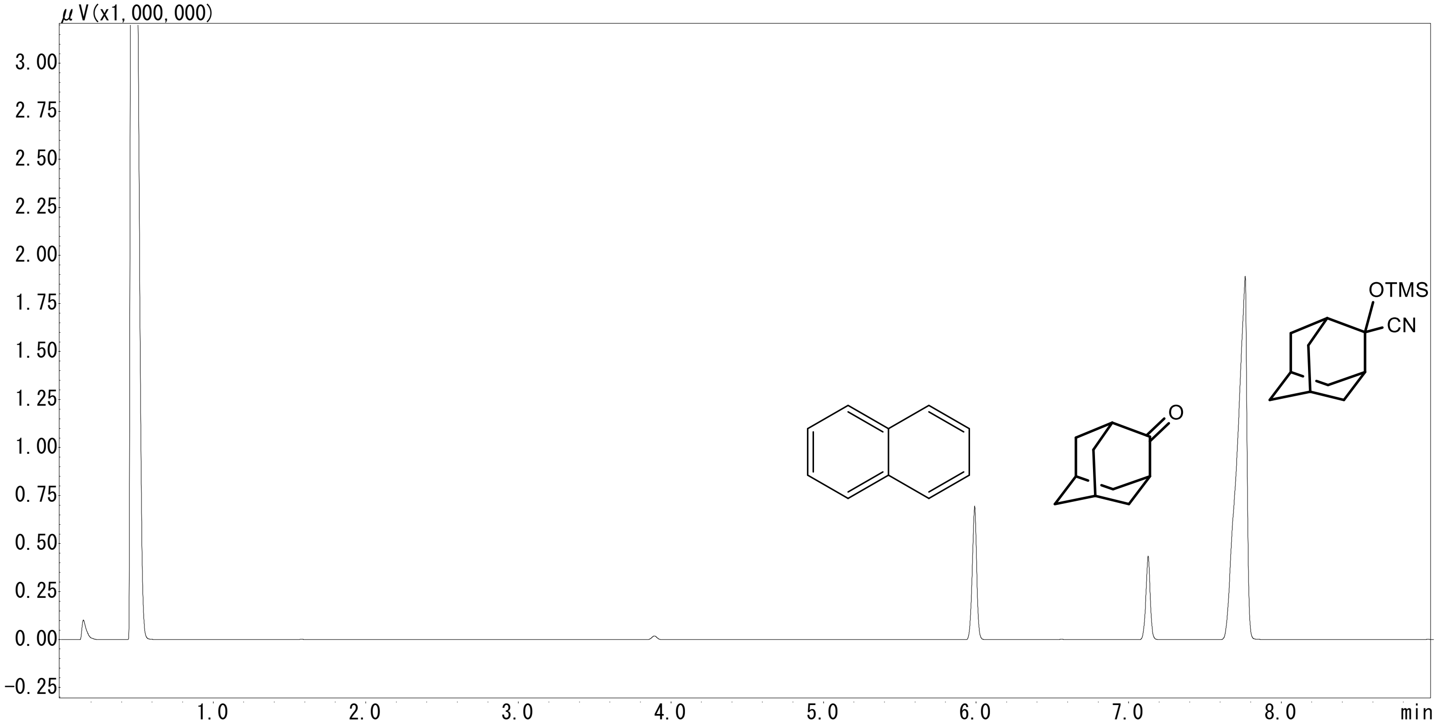

MS spectrum

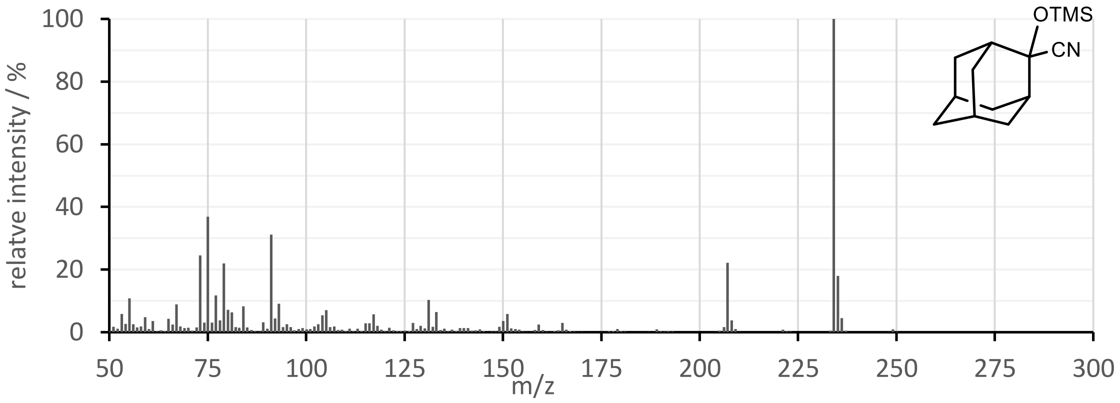

GC chart

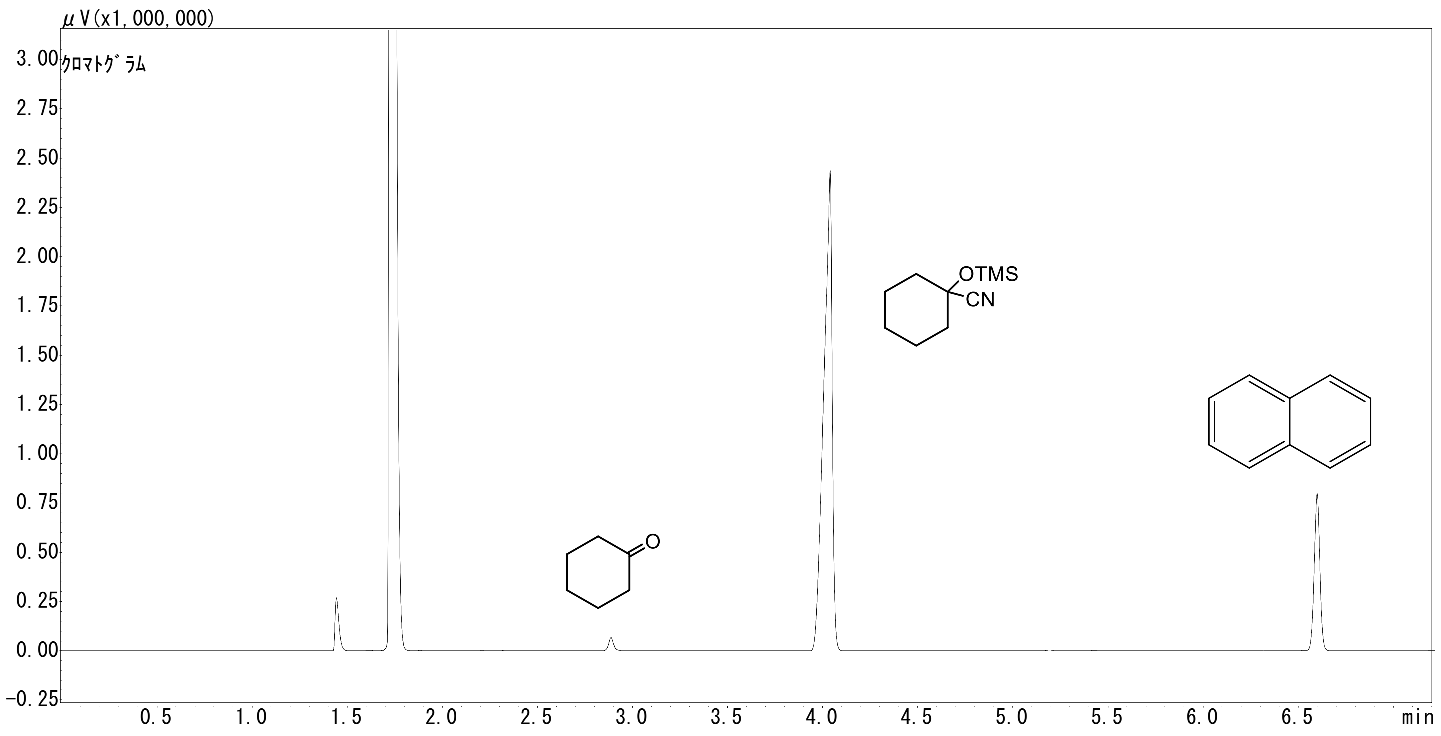

MS spectrum

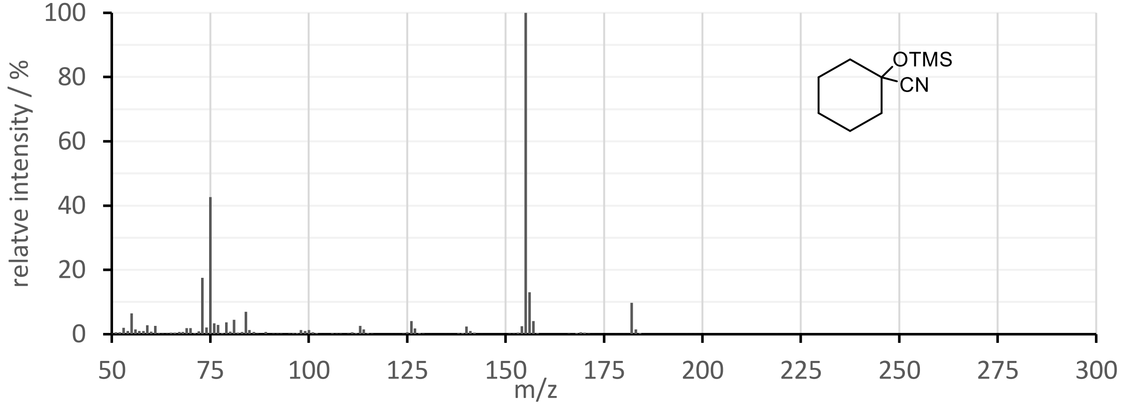

GC chart

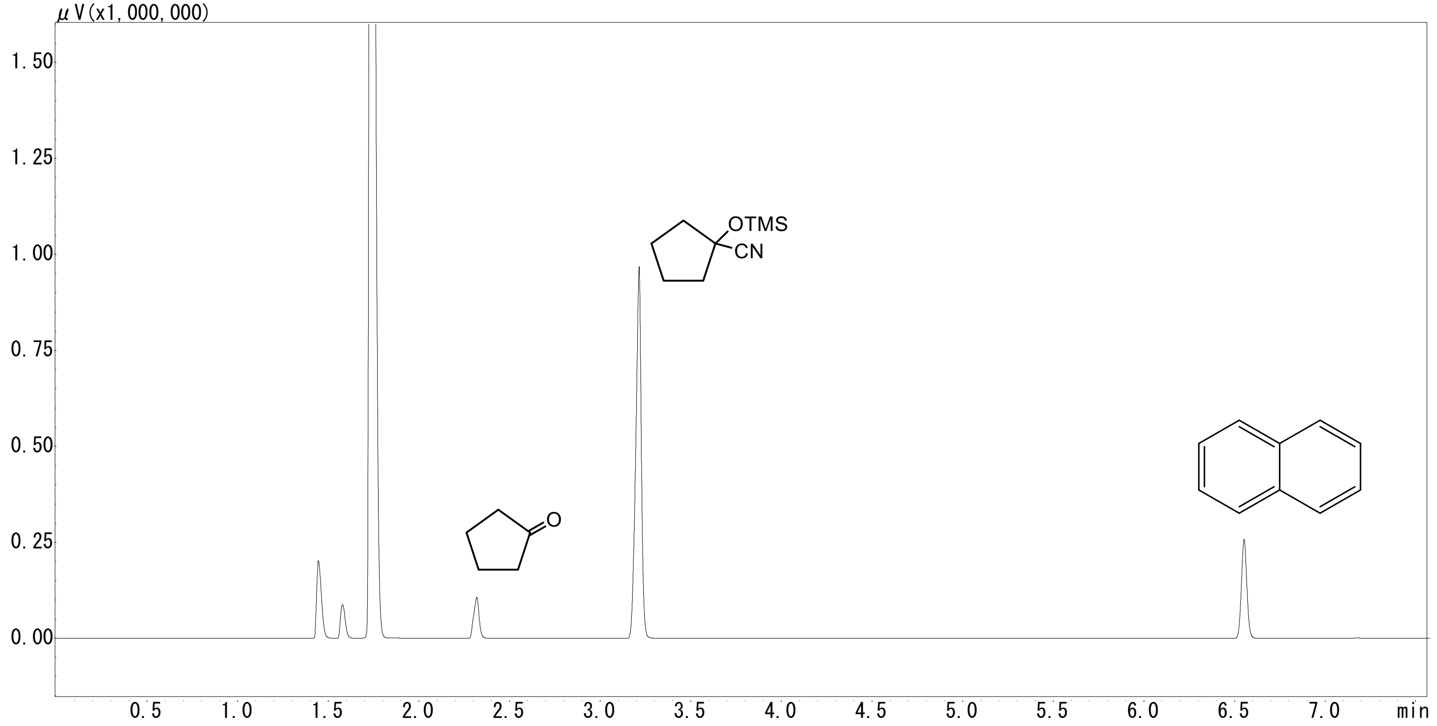

MS spectrum

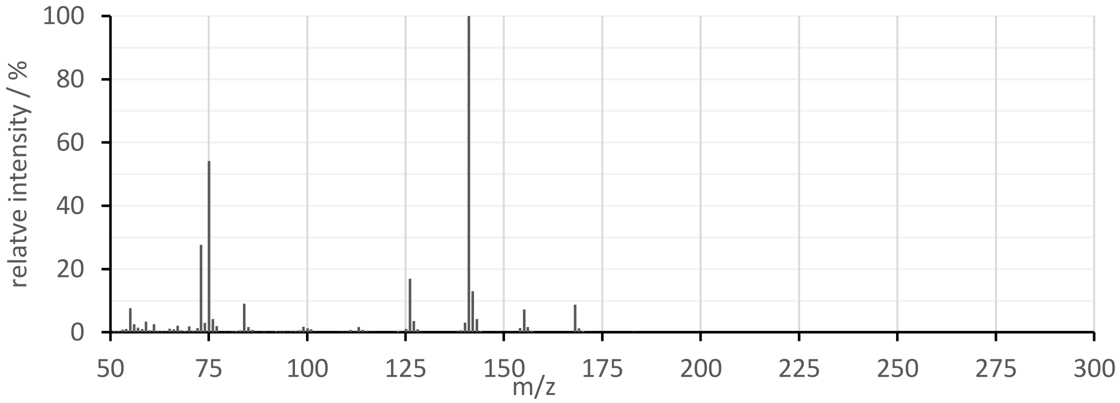

GC chart

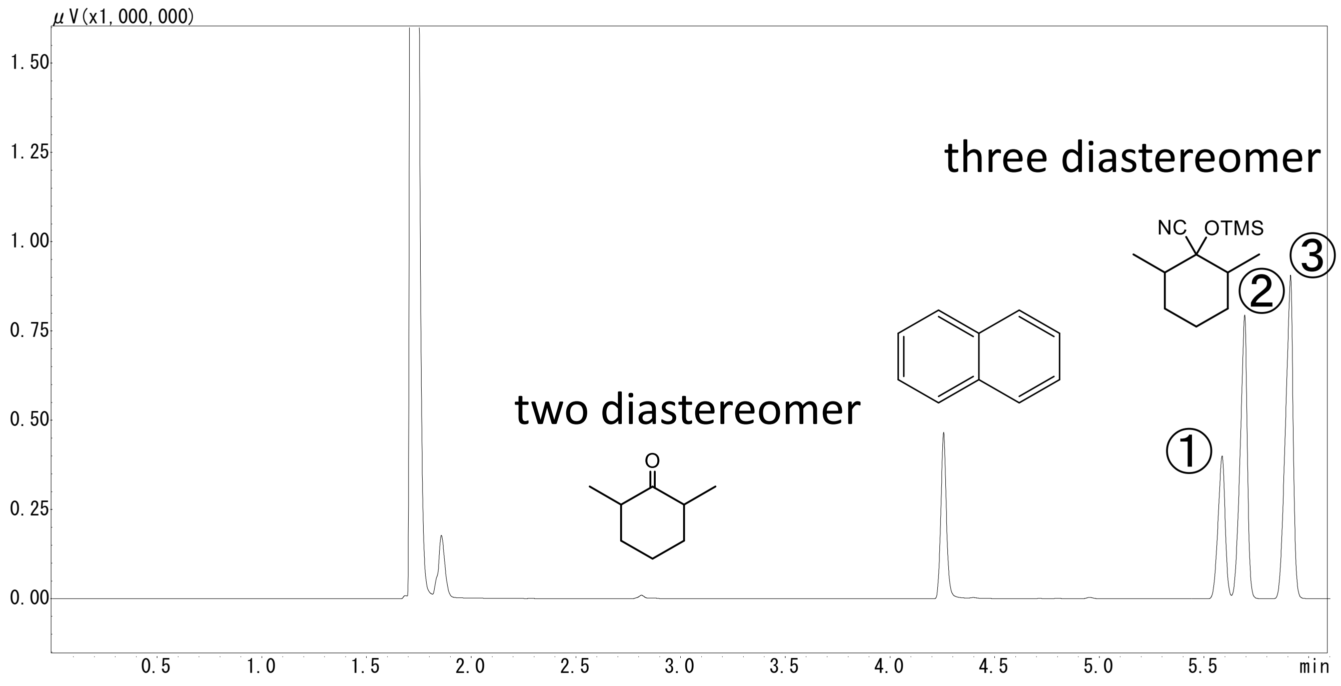

MS spectrum

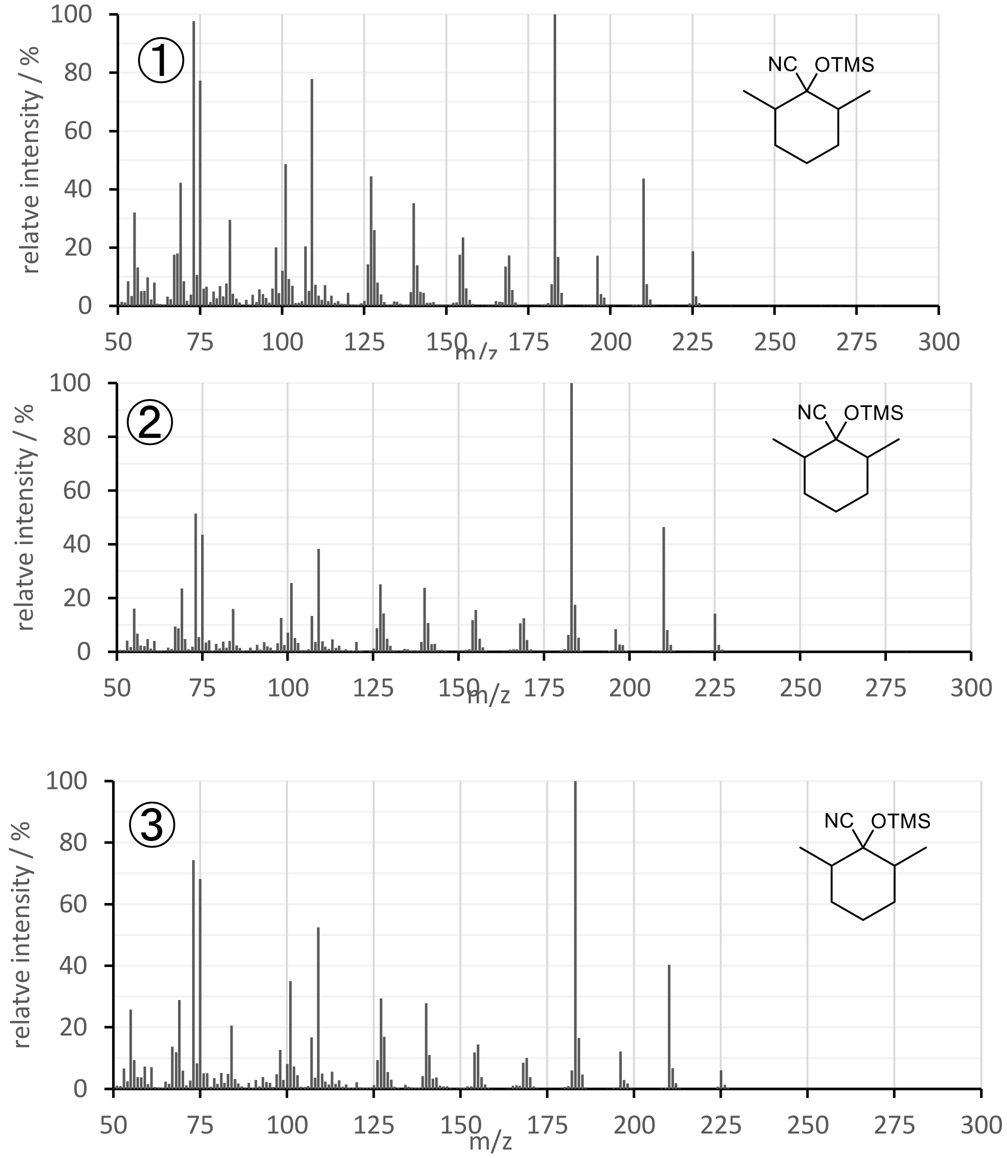

GC chart

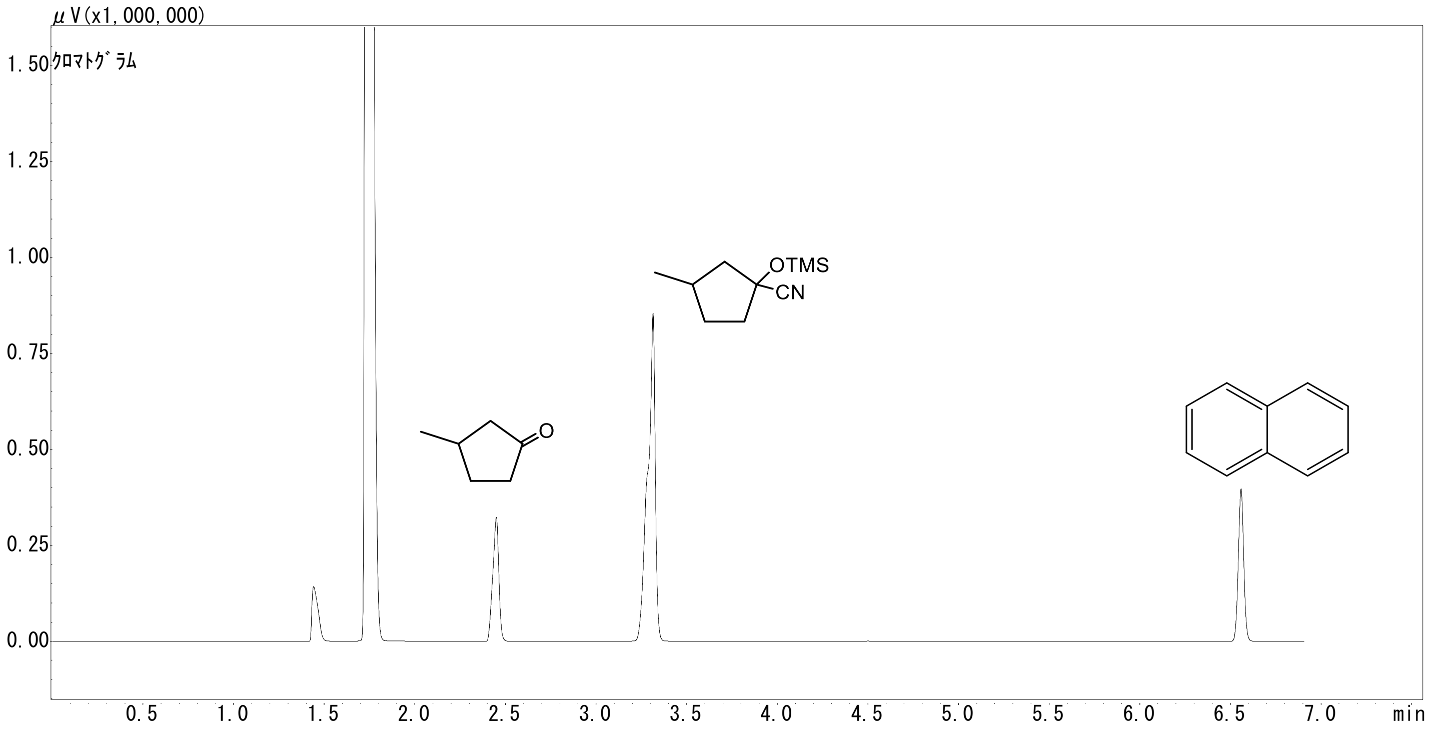

MS spectrum

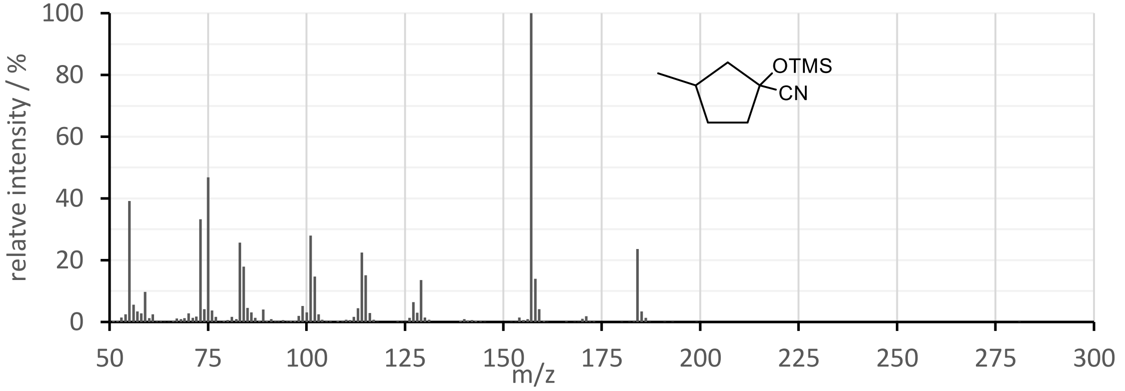

GC chart

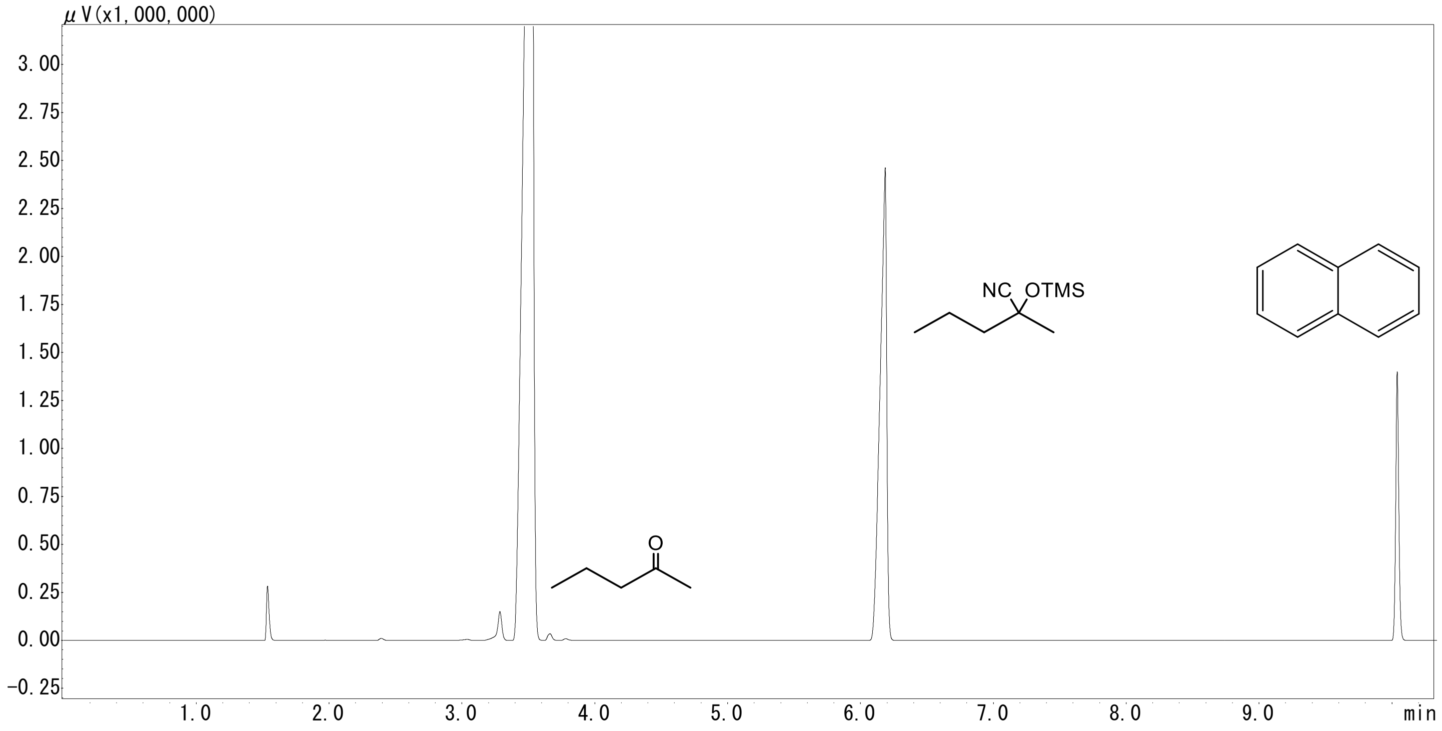

MS spectrum

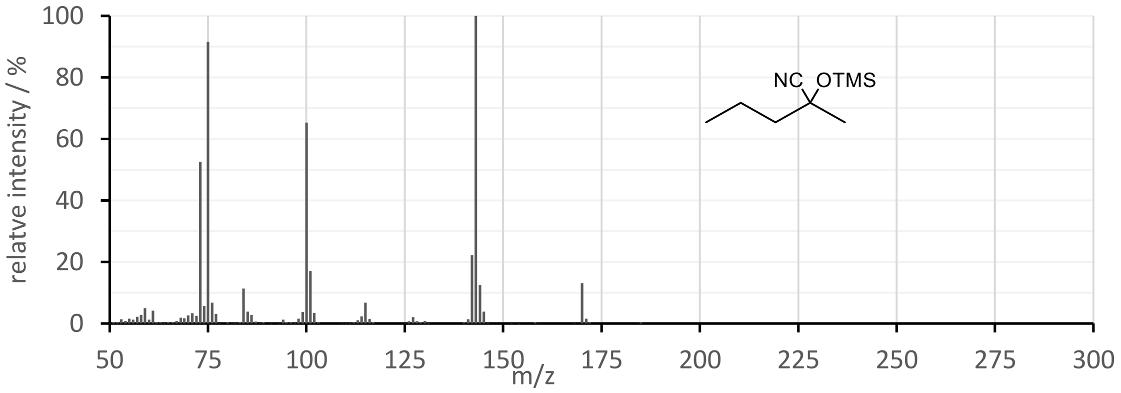

GC chart

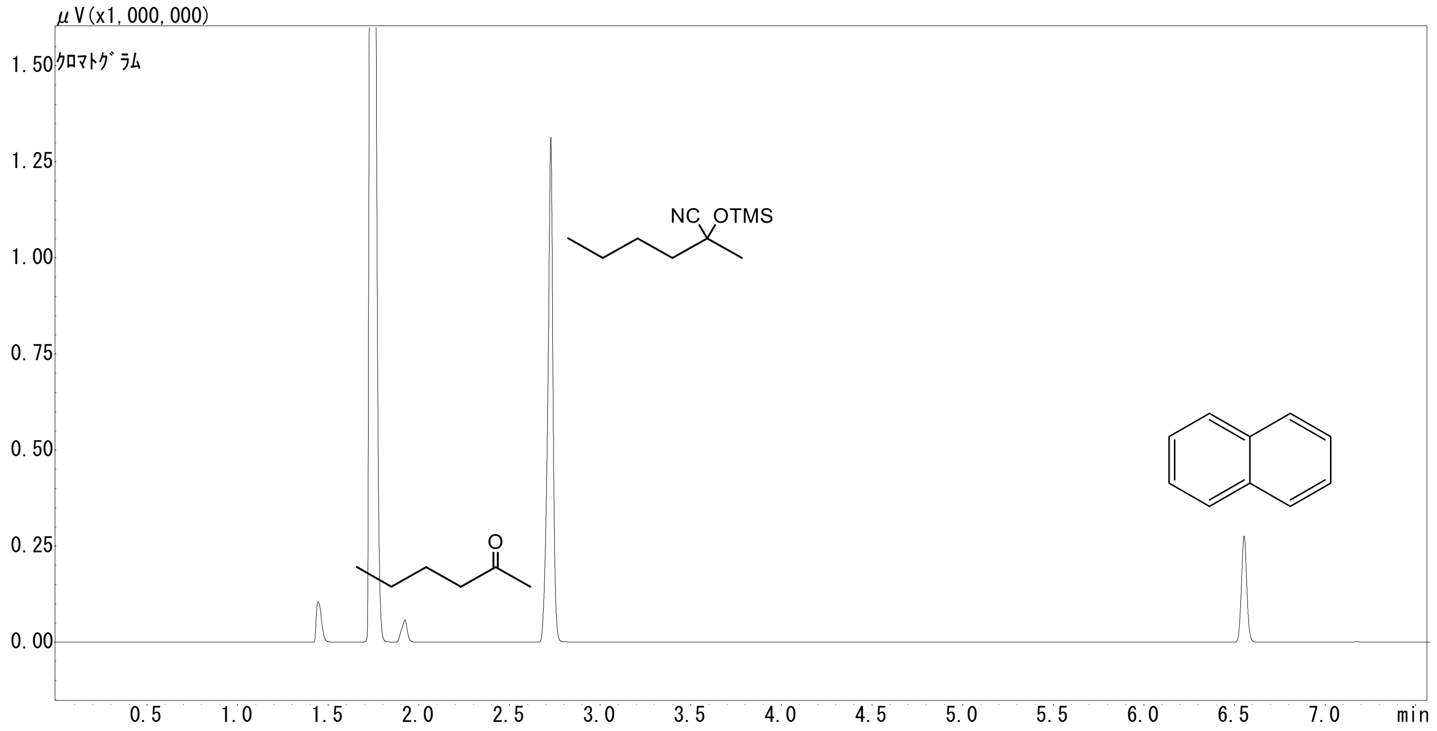

MS spectrum

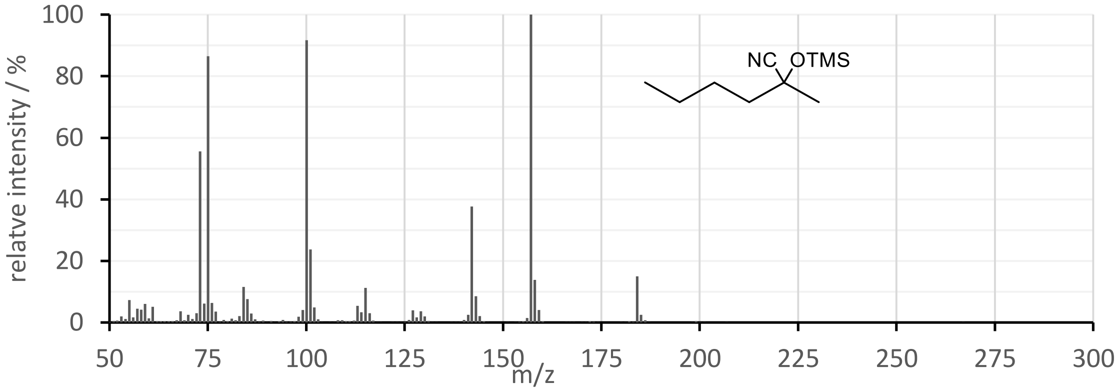

GC chart

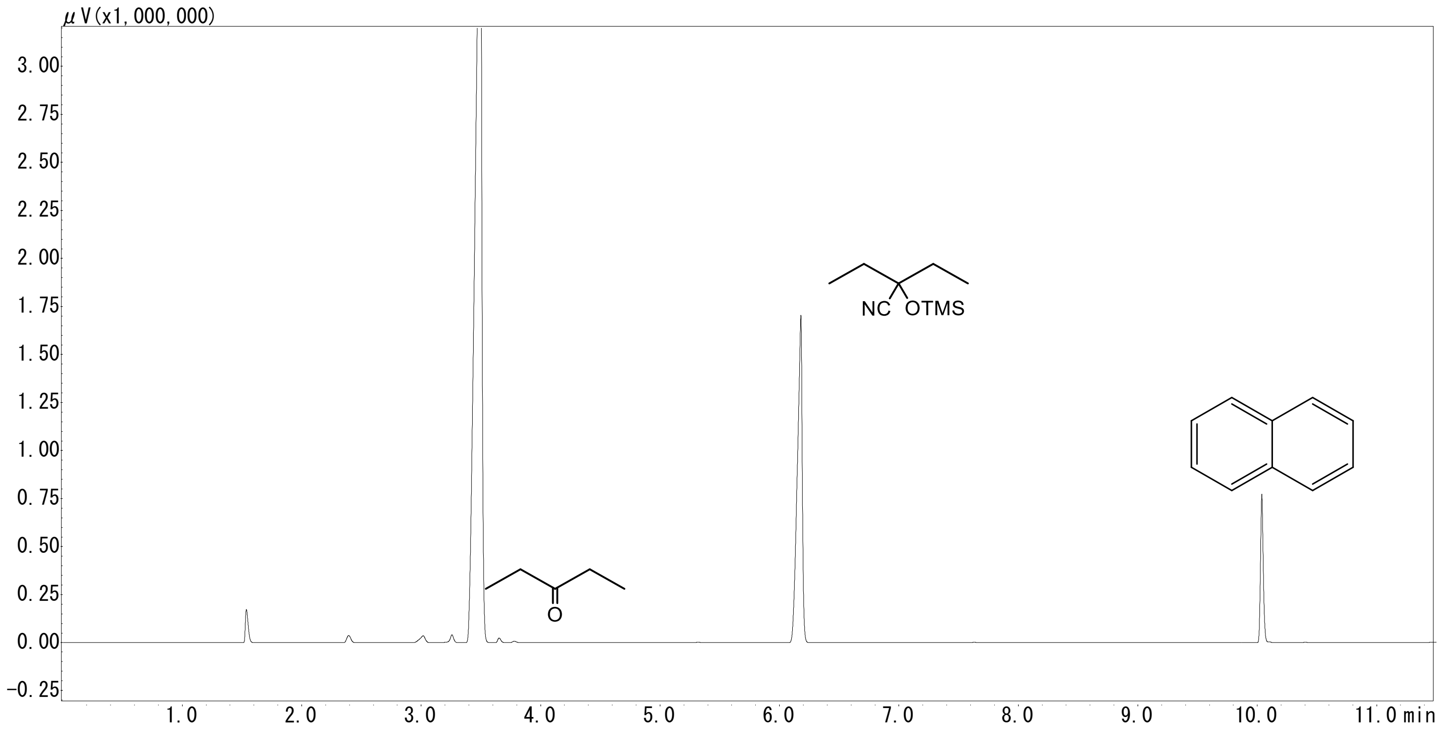

MS spectrum

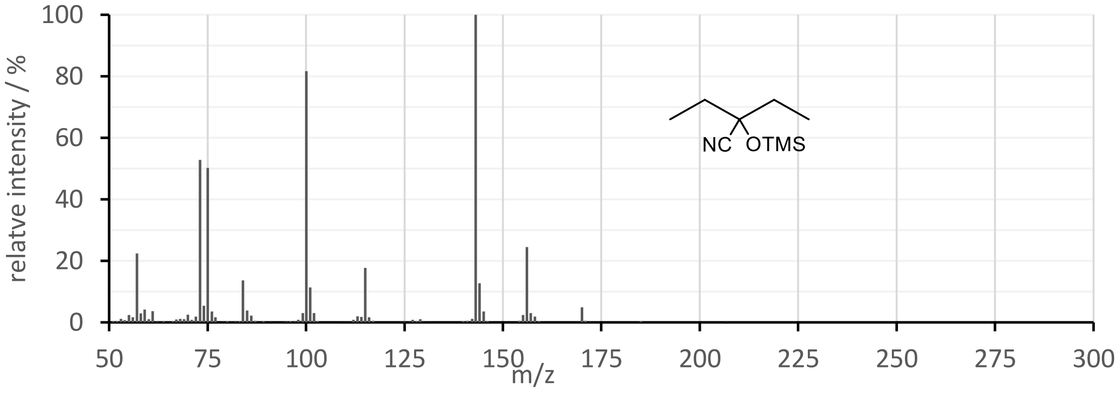

GC chart

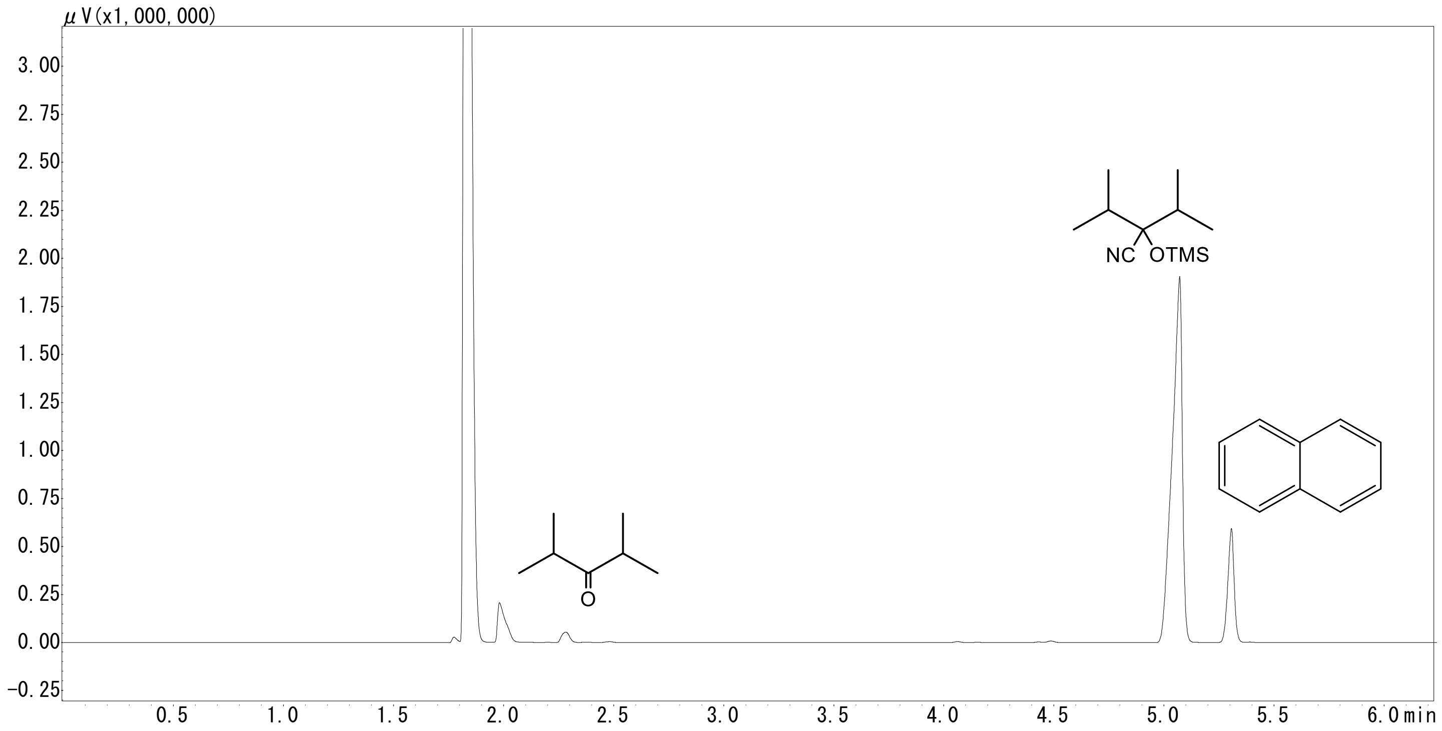

MS spectrum

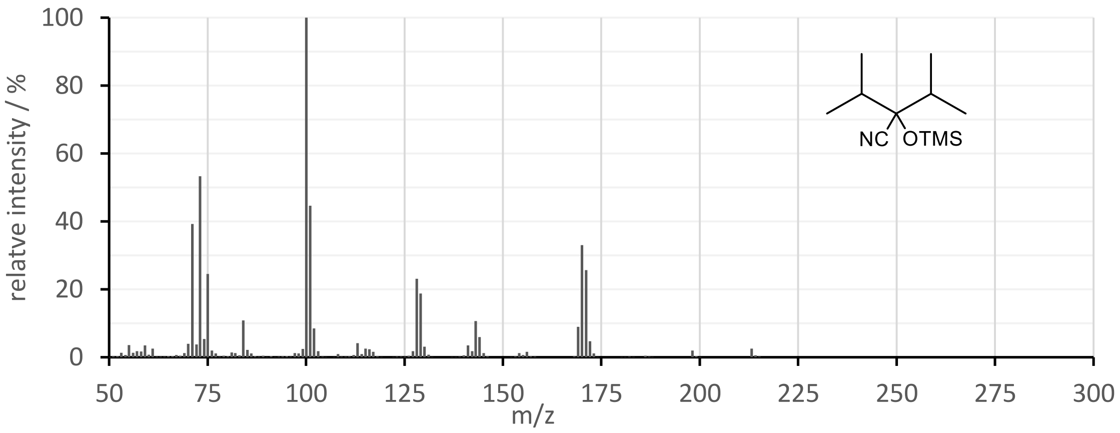

GC chart

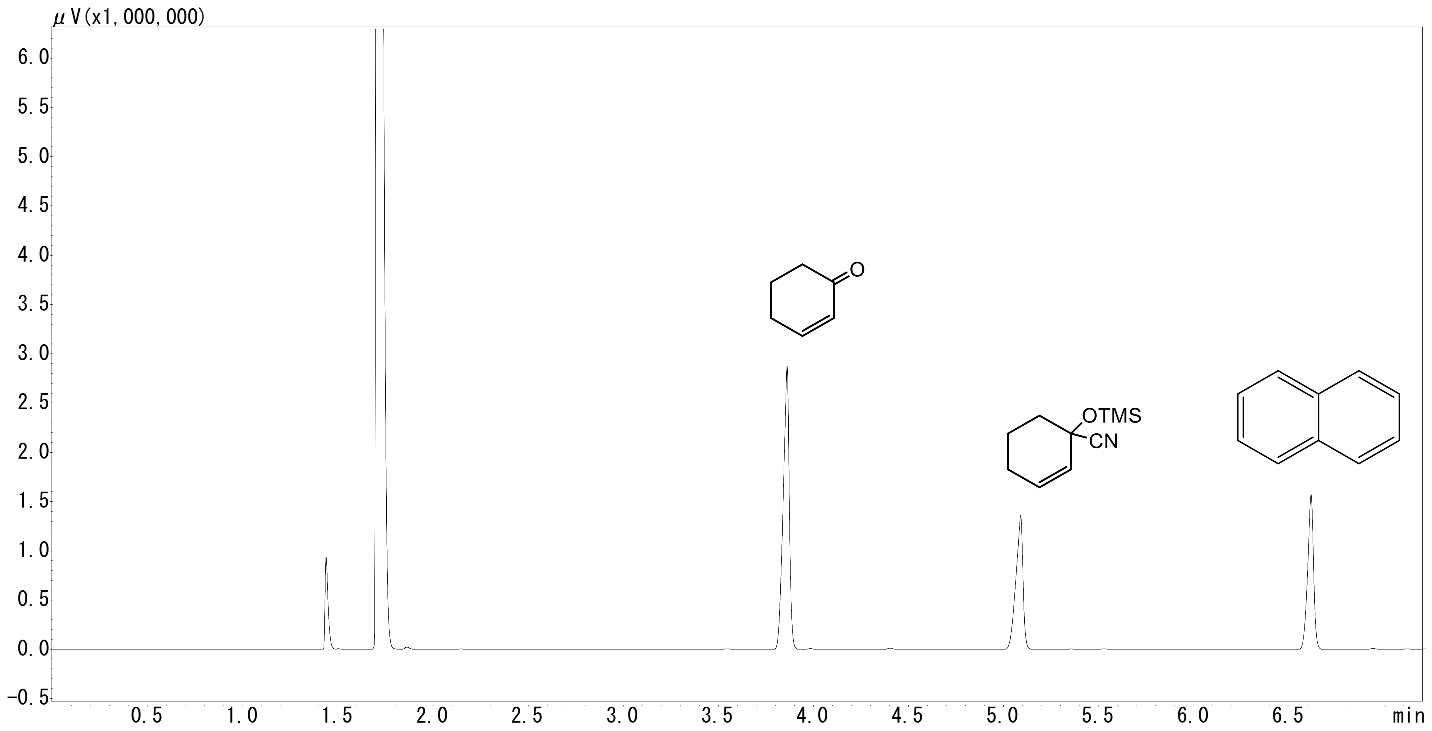

MS spectrum

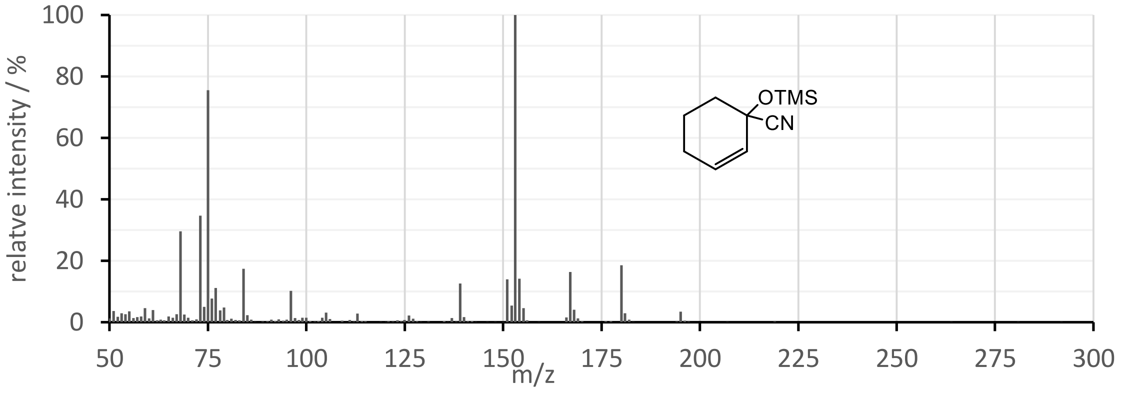

GC chart

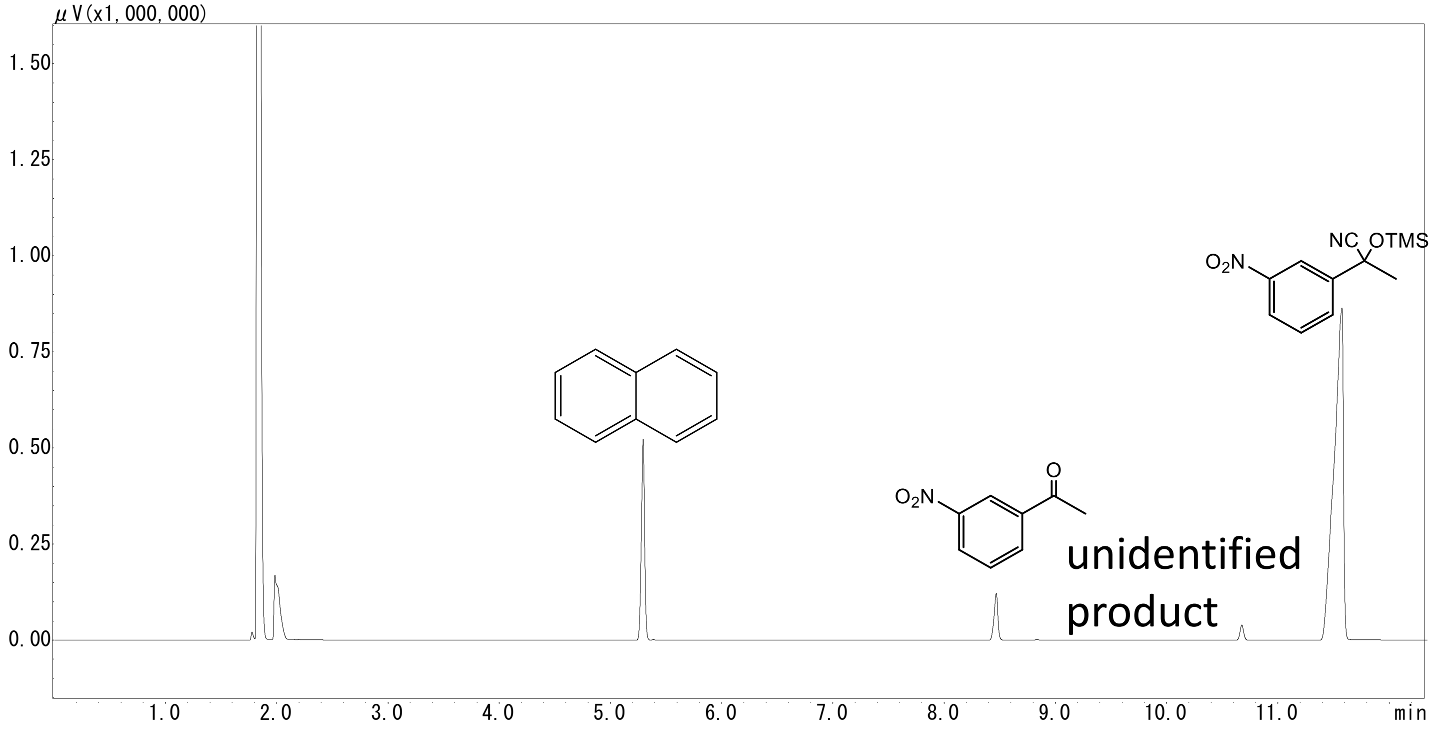

MS spectrum

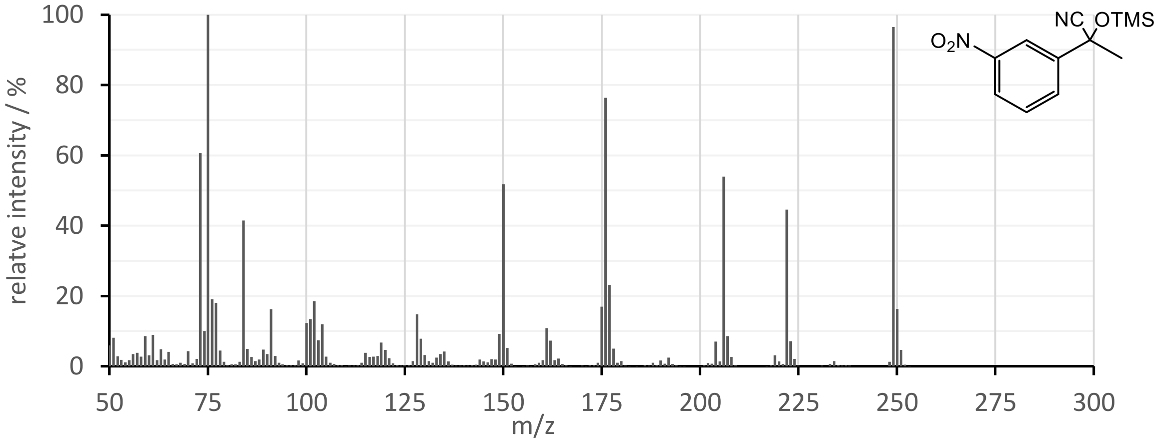

GC chart

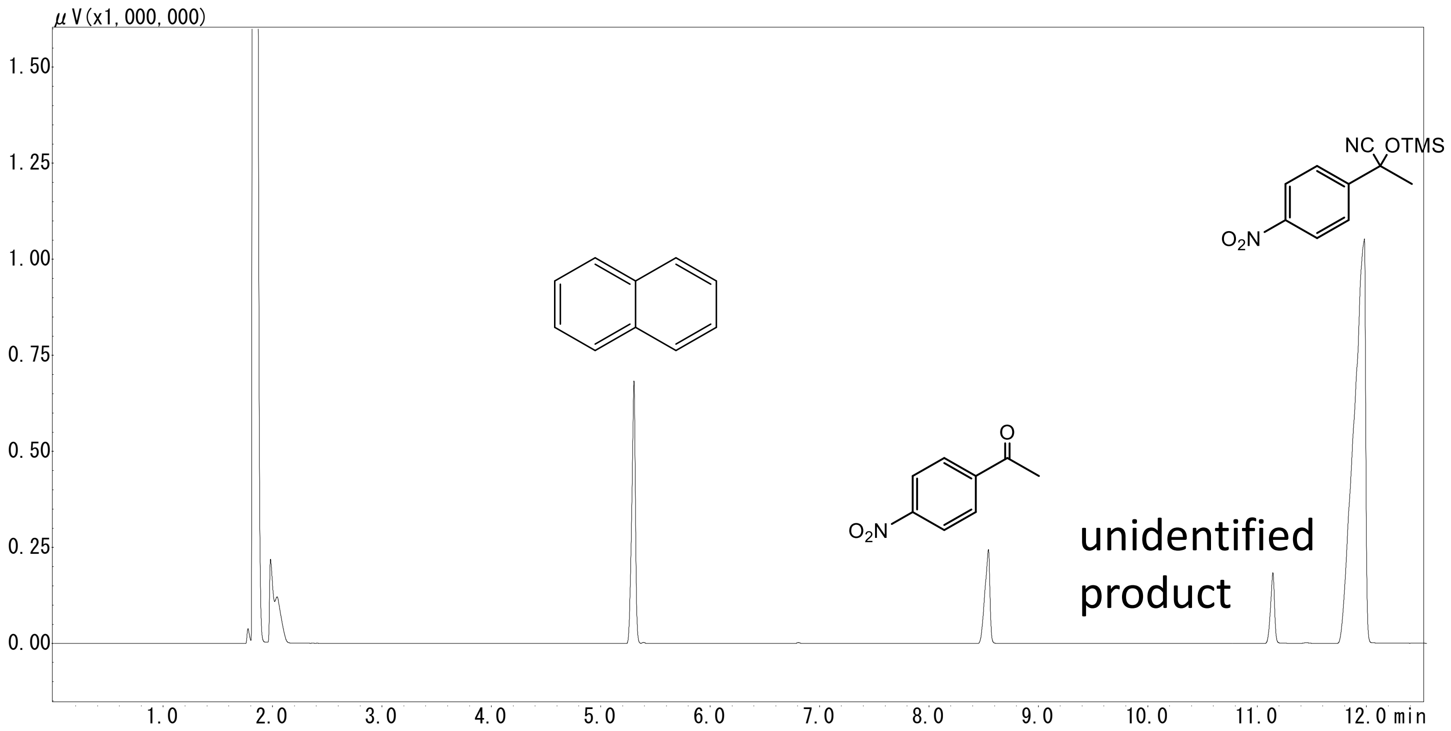

MS spectrum

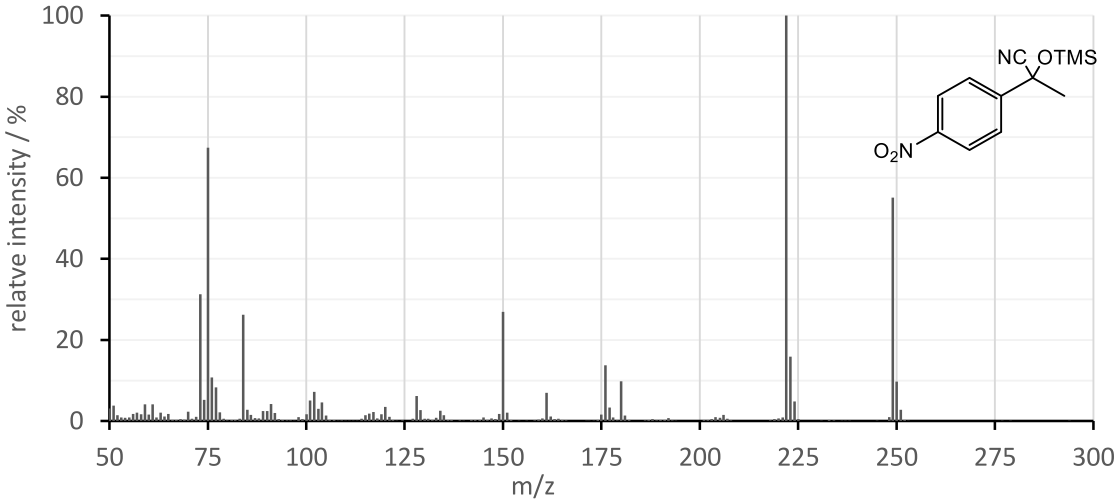

GC chart

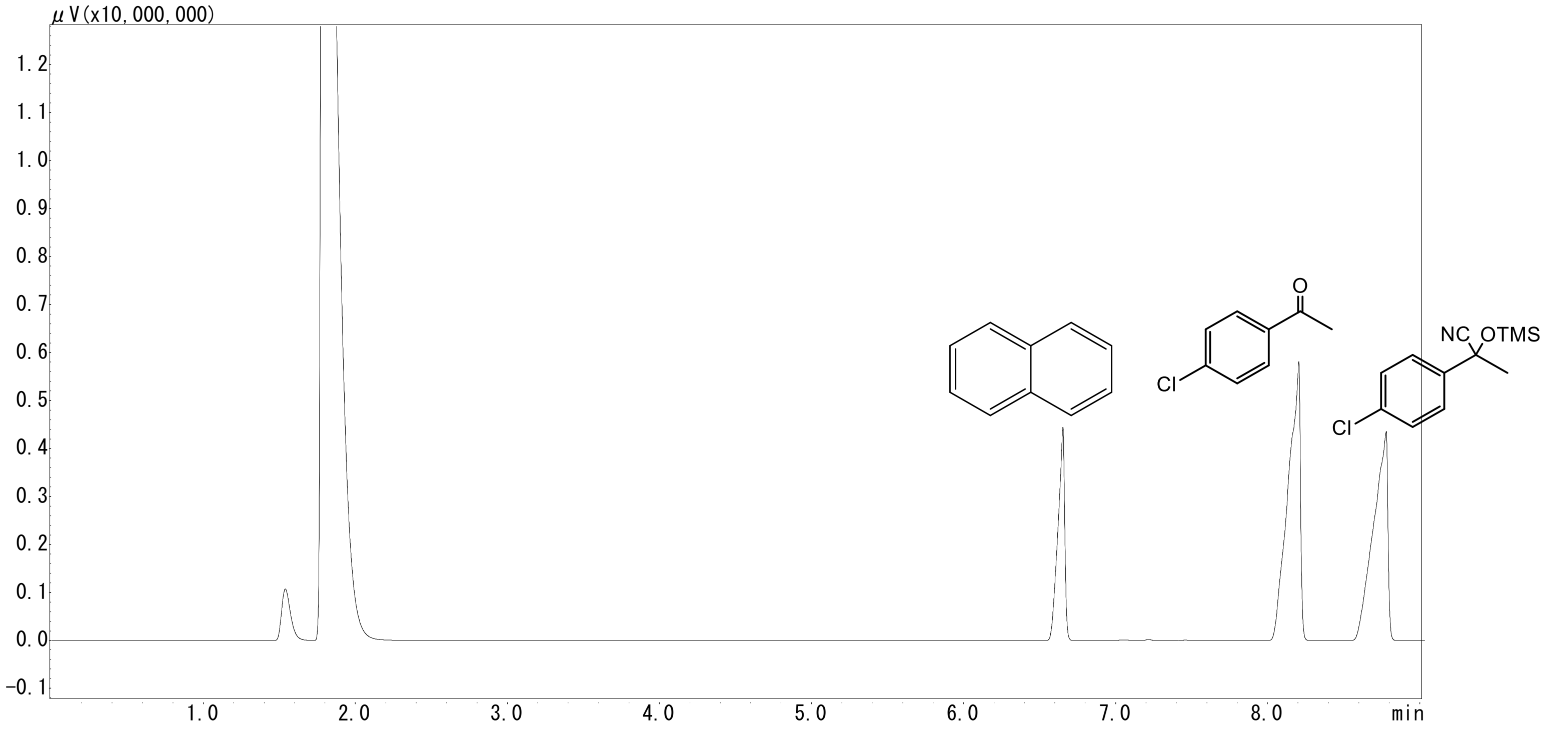

MS spectrum

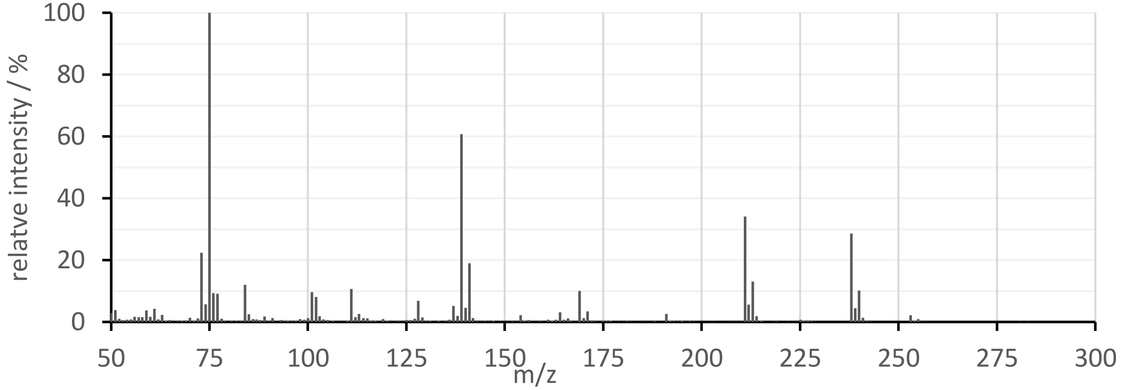

GC chart

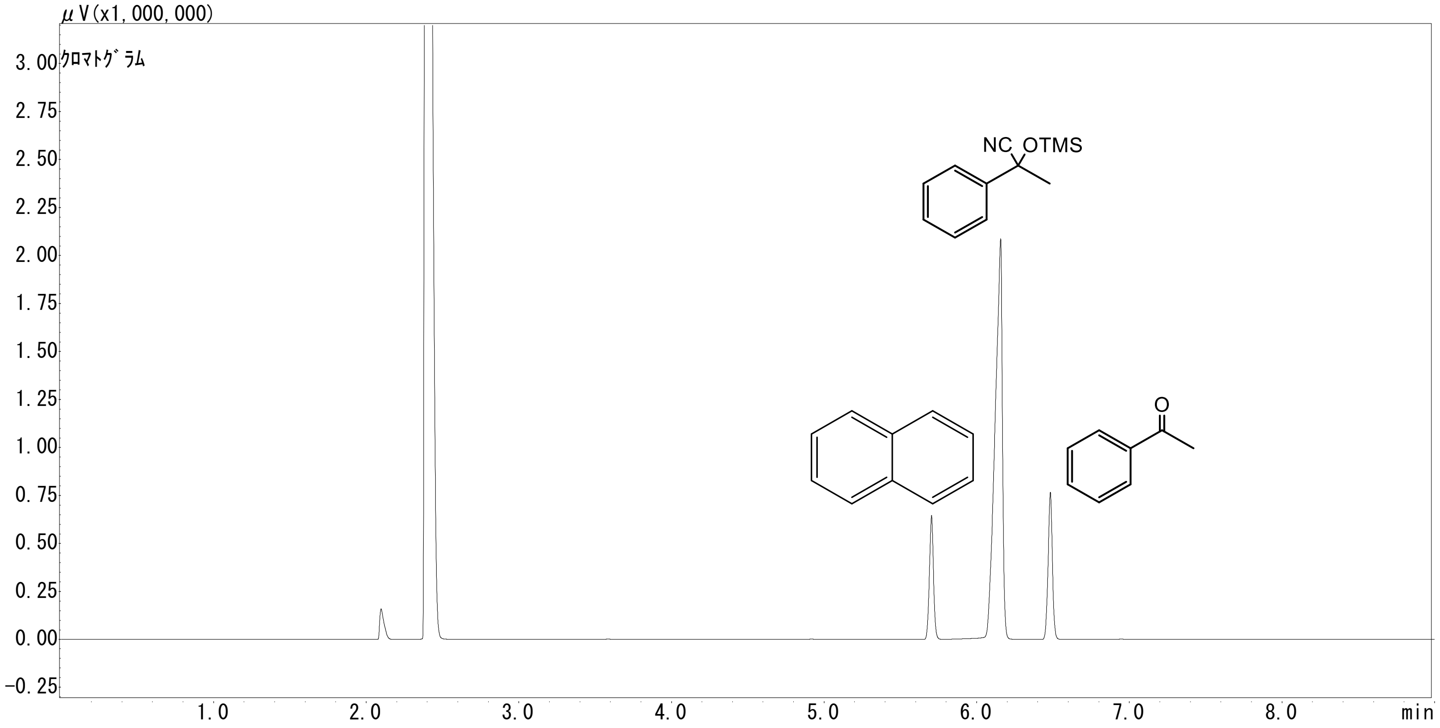

MS spectrum

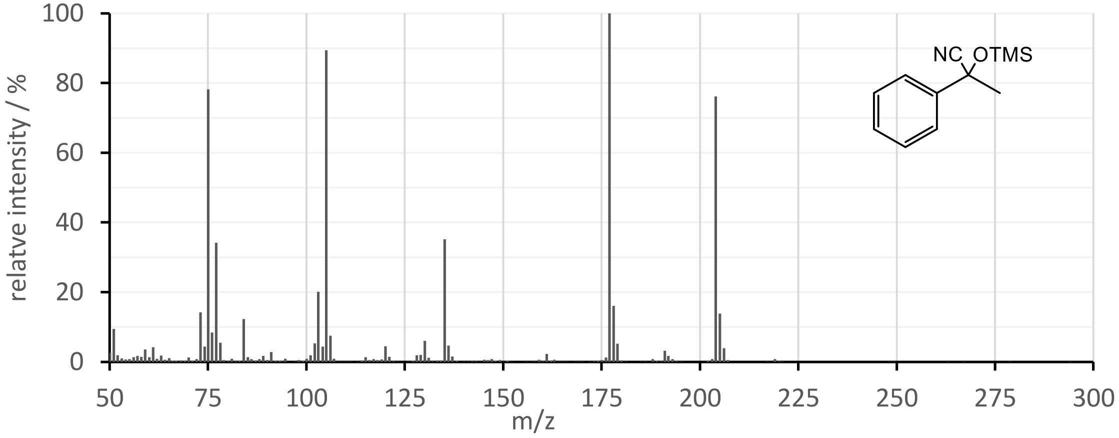

GC chart

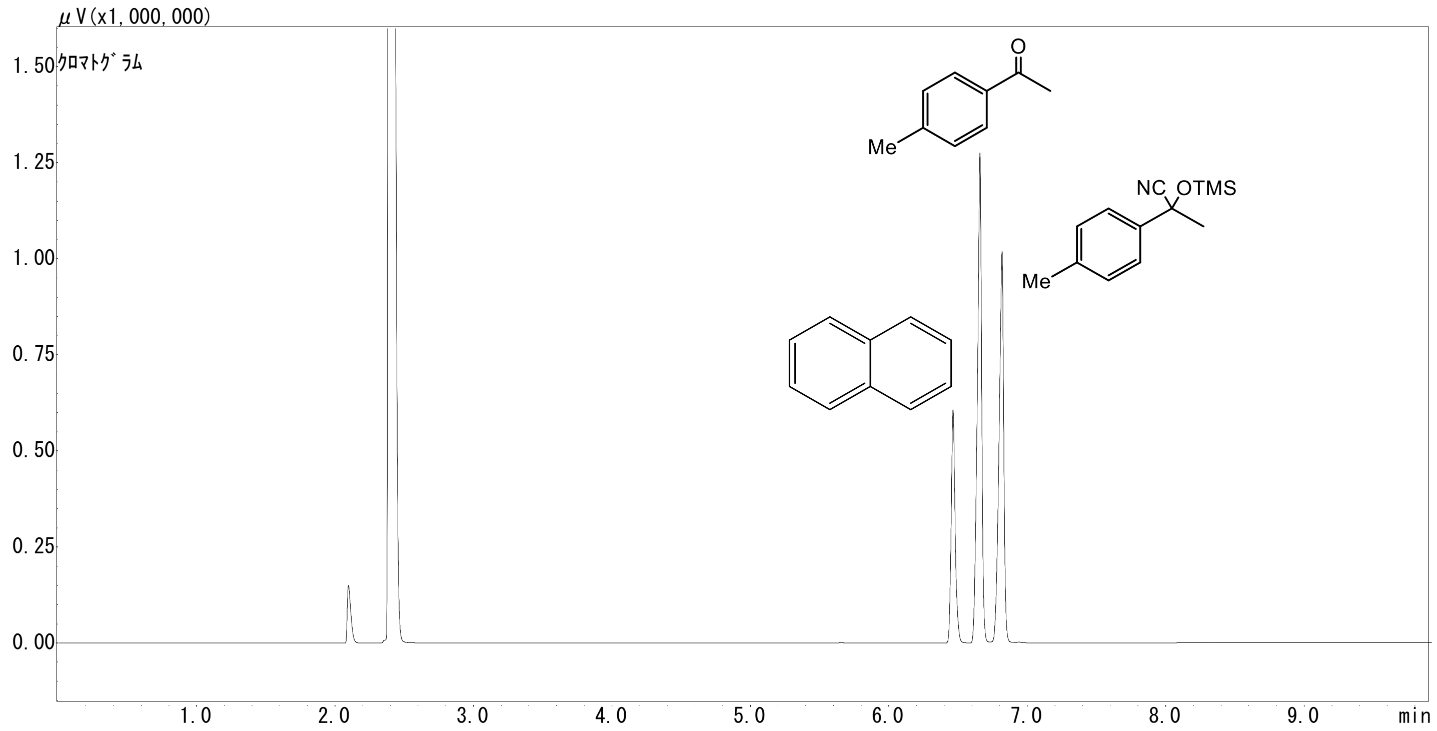

MS spectrum

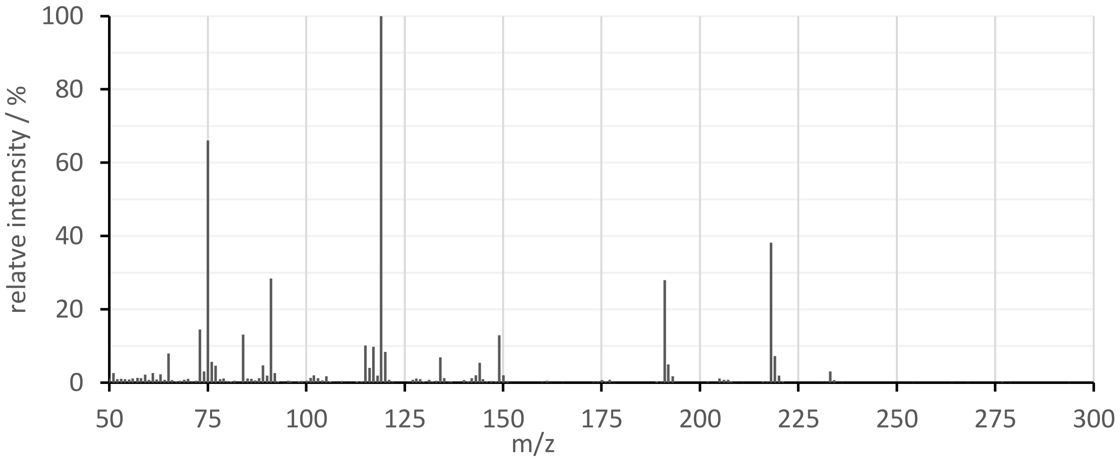

GC chart

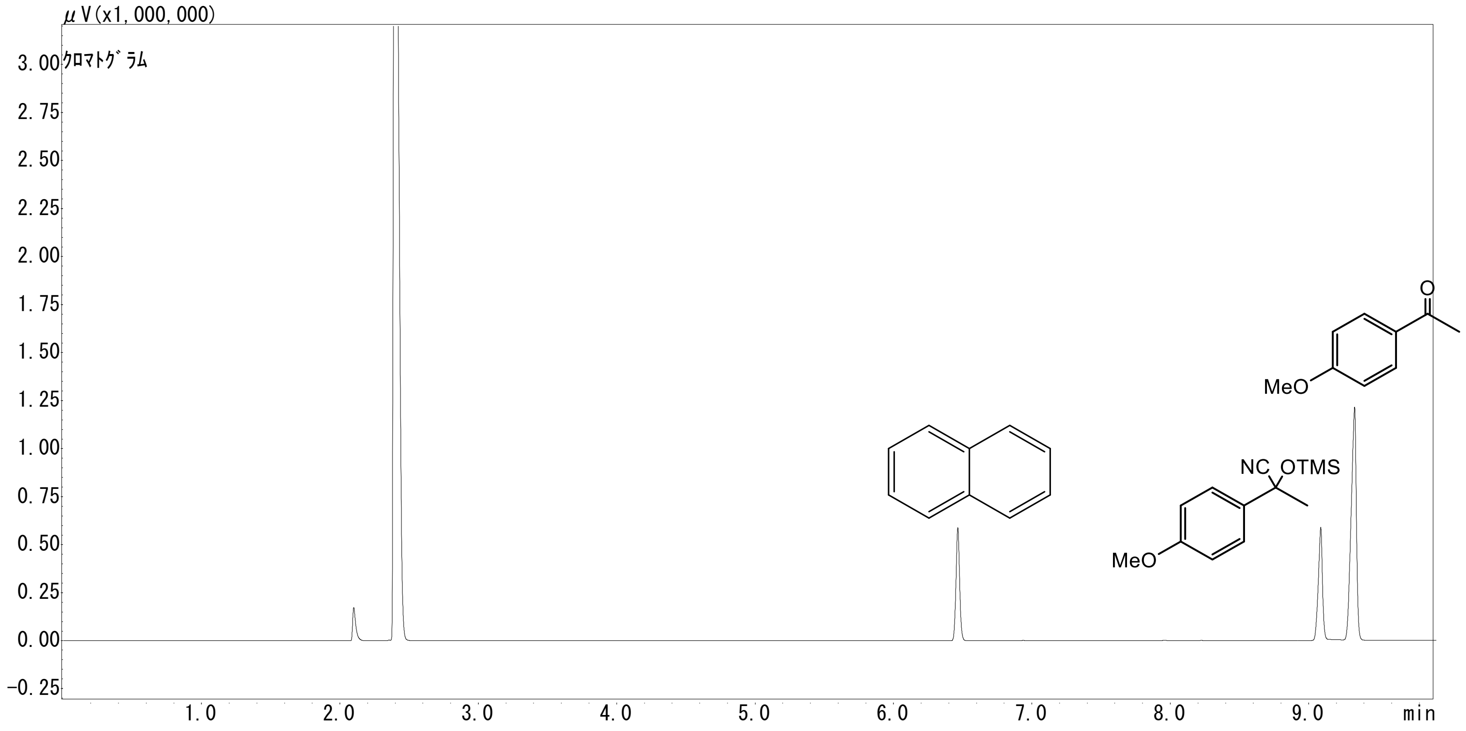

MS spectrum

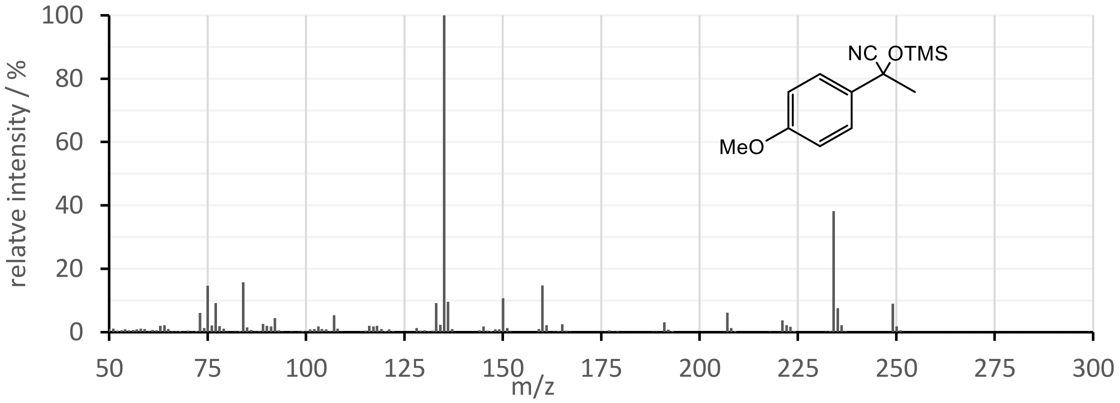

GC chart

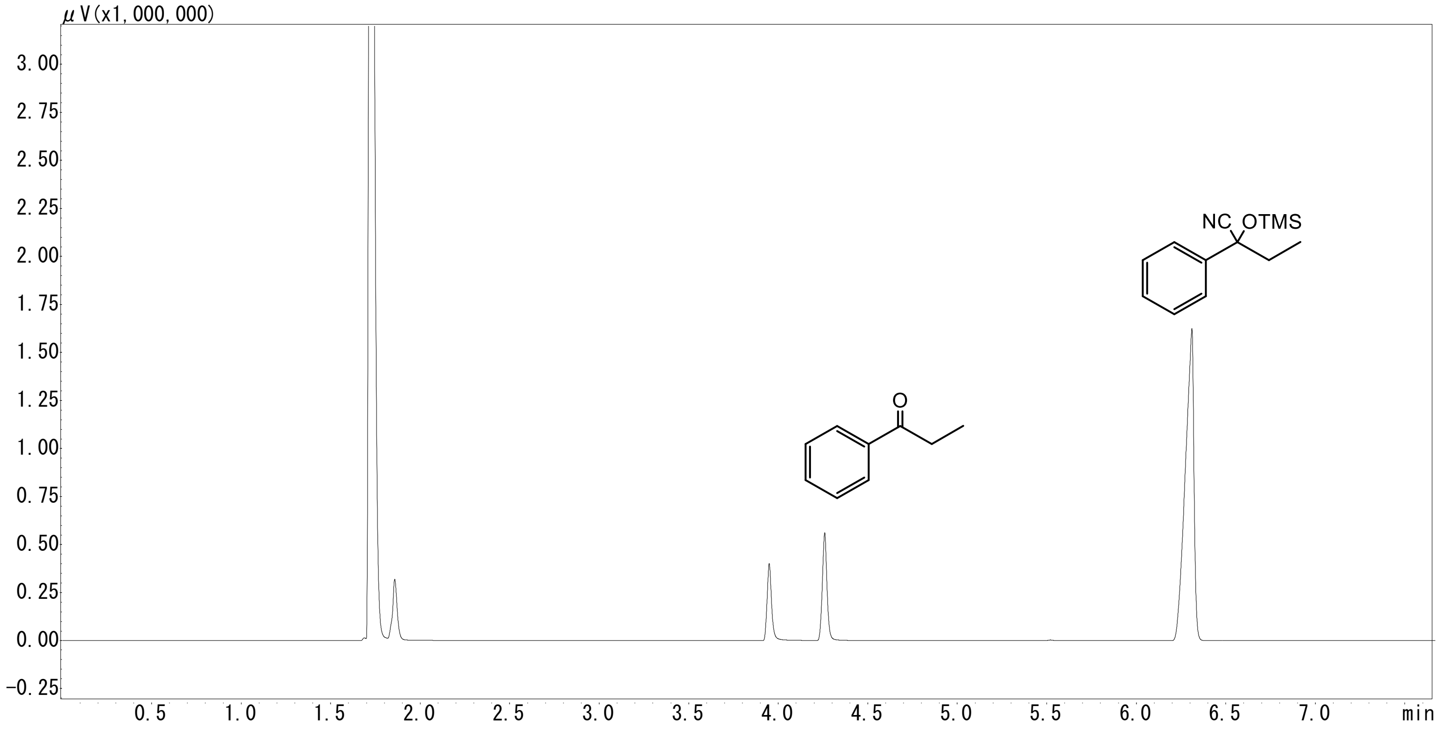

MS spectrum

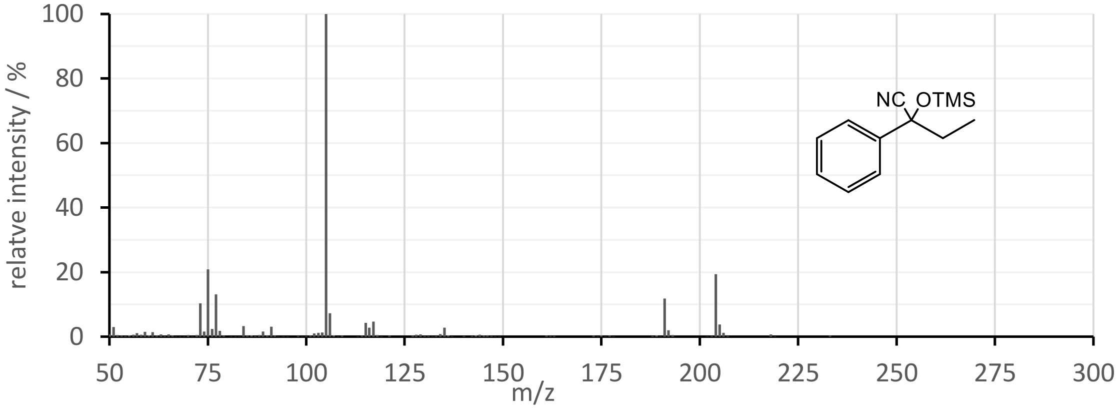

GC chart

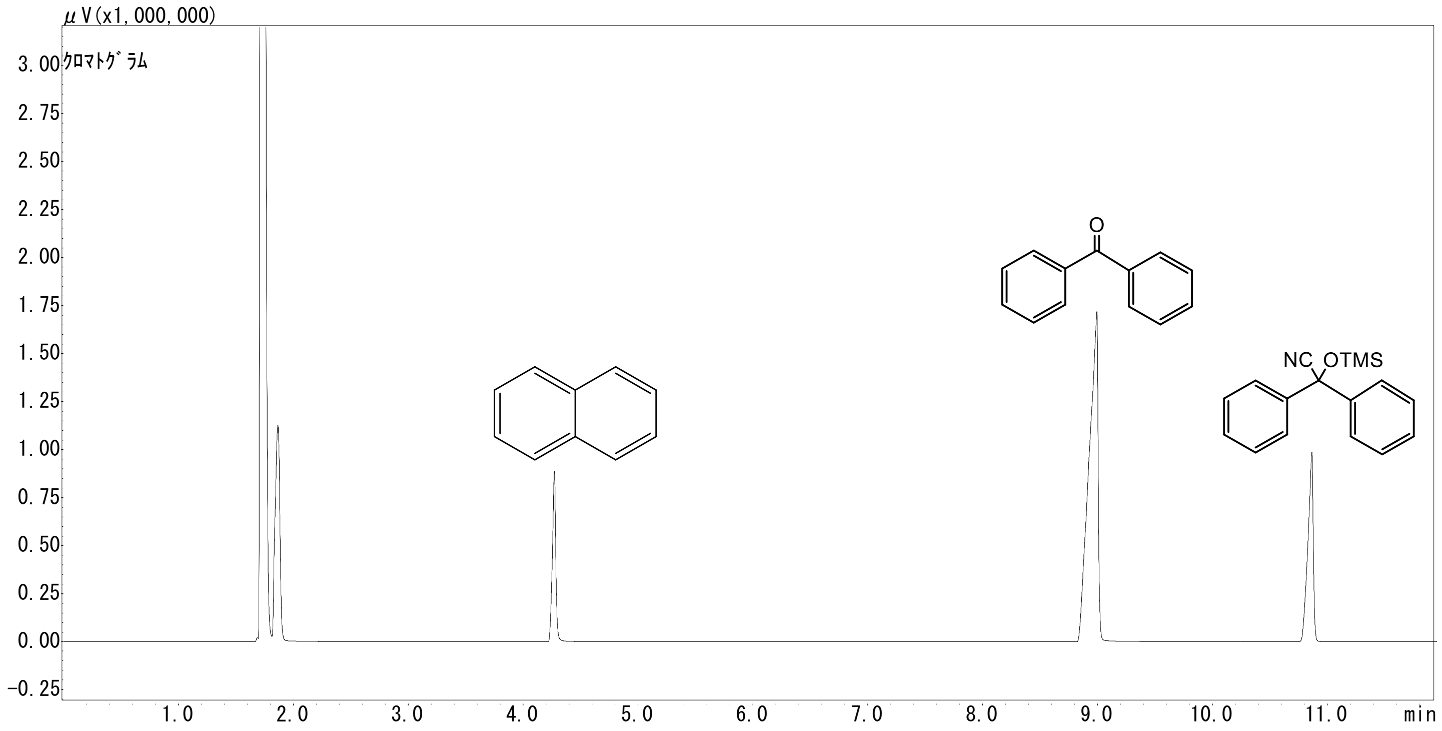

MS spectrum

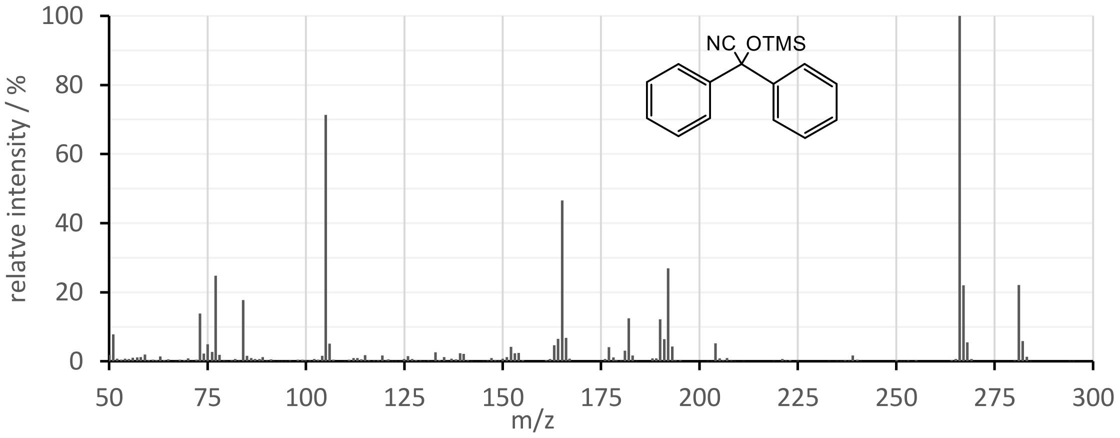

GC chart

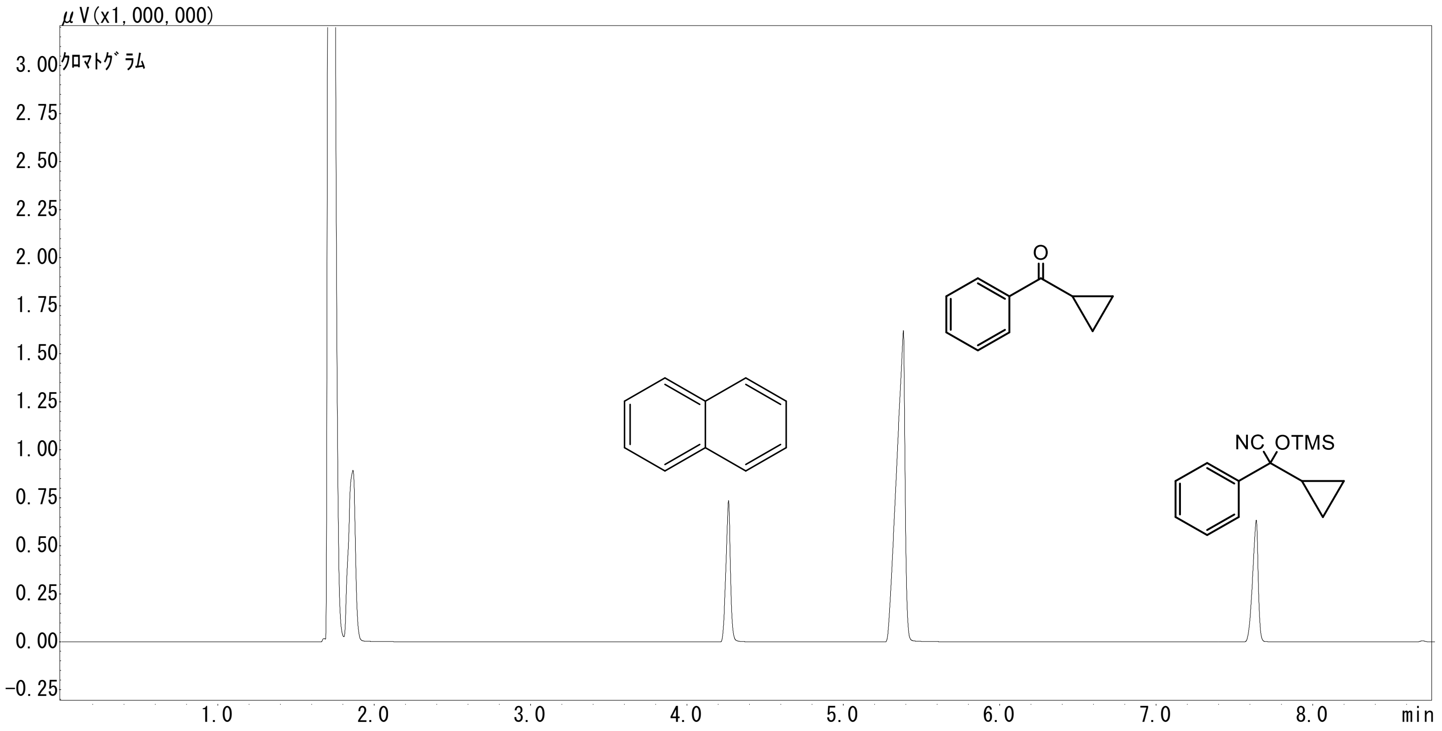

MS spectrum

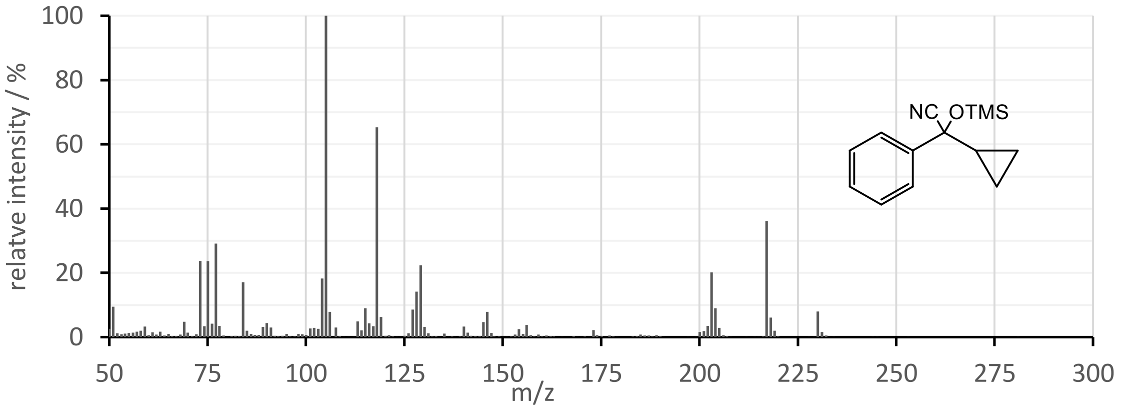

GC chart

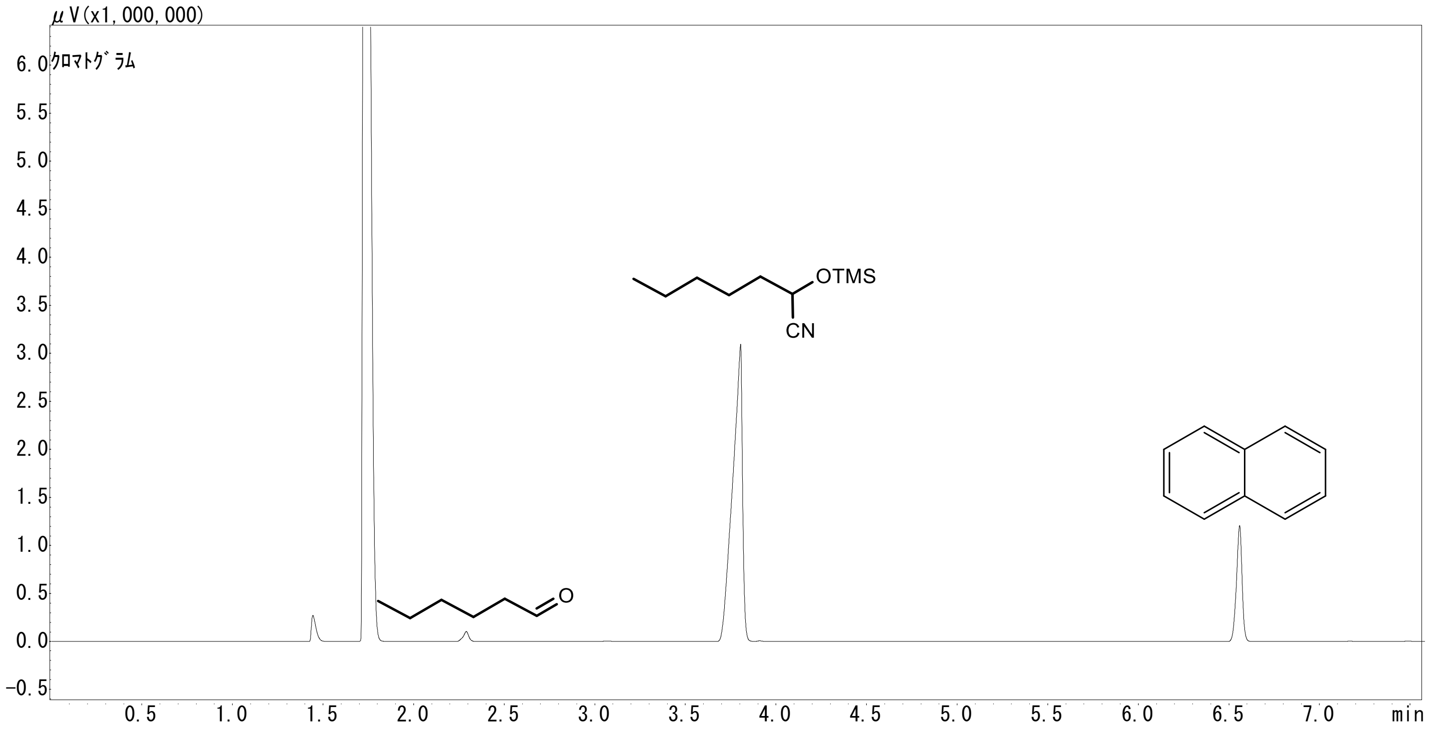

MS spectrum

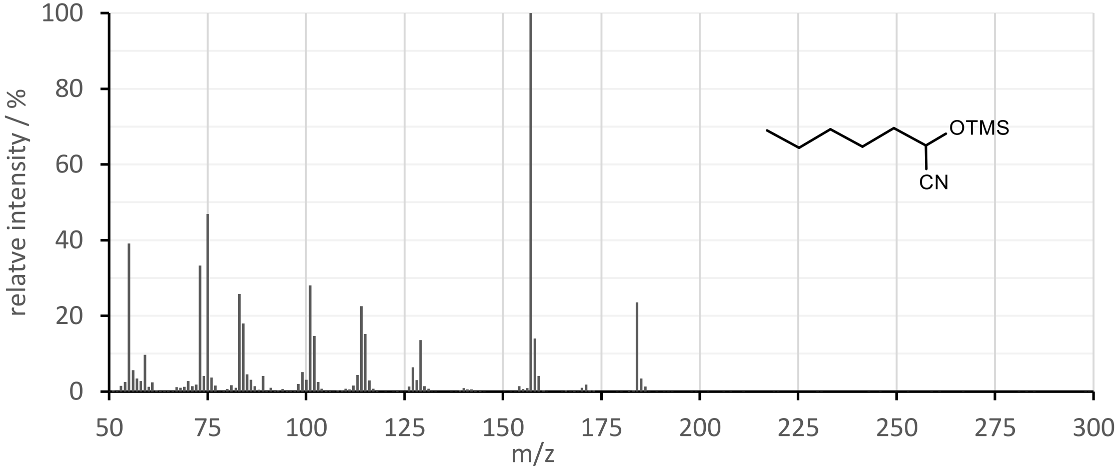

GC chart

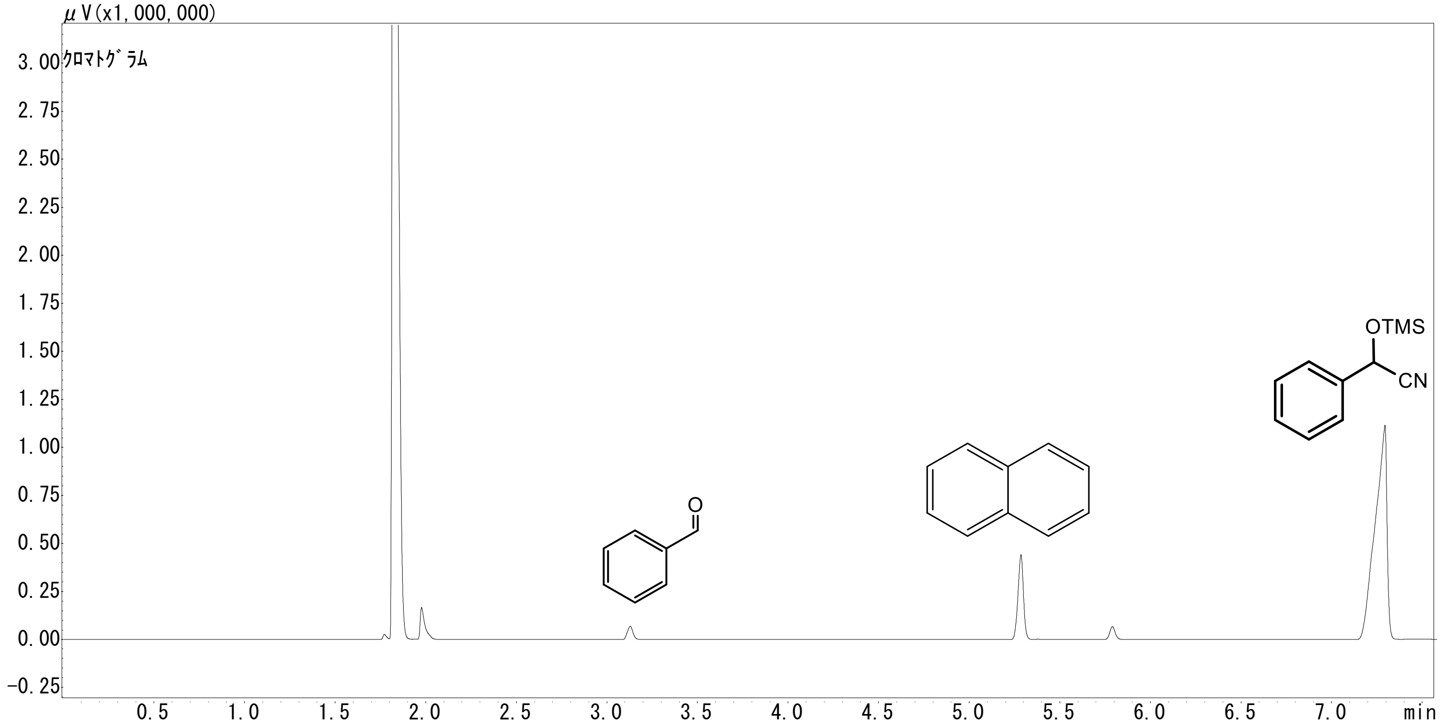

MS spectrum

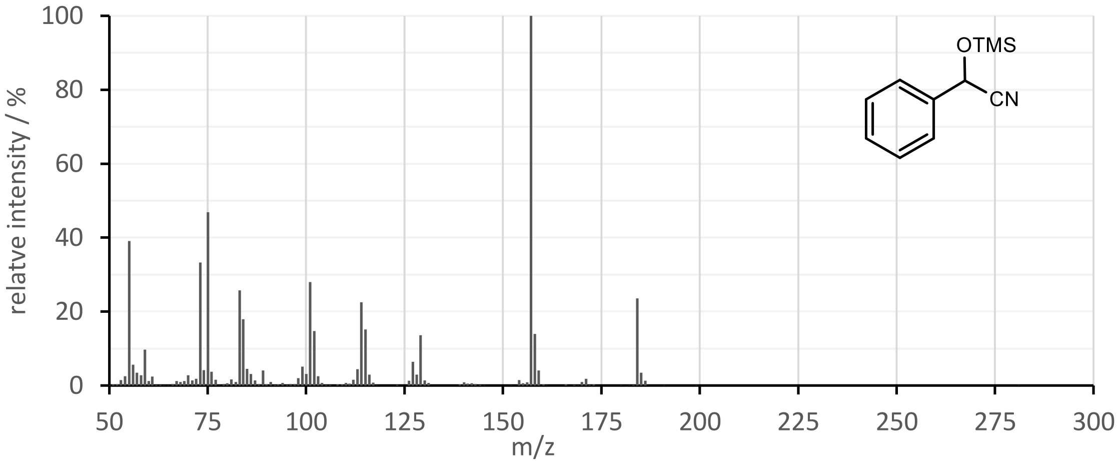

Supplement: RA-011-D1RA05879G-s002 [file RA-011-D1RA05879G-s002.pdf]
